# Exploring the links between peptoid antibacterial activity and toxicity†
†The authors declare no competing interests.
‡
‡Electronic supplementary information (ESI) available. See DOI: 10.1039/c6md00648e


**DOI:** 10.1039/c6md00648e

**Published:** 2017-02-01

**Authors:** H. L. Bolt, G. A. Eggimann, C. A. B. Jahoda, R. N. Zuckermann, G. J. Sharples, S. L. Cobb

**Affiliations:** a Department of Chemistry , Durham University , South Road , Durham , DH1 3LE , UK . Email: s.l.cobb@durham.ac.uk; b School of Biological and Biomedical Sciences , Durham University , Durham DH1 3LE , UK . Email: gary.sharples@durham.ac.uk; c Molecular Foundry , Lawrence Berkeley National Laboratory , Berkeley , California , USA

## Abstract

Peptoids are a promising class of antimicrobial agents with reported activities against a range of both Gram-positive and Gram-negative bacteria, fungi and most recently parasites. However, at present the available toxicity data is somewhat limited and as such rationally designing effective antimicrobial peptoids can be challenging. Herein, we present the toxicity profiling of a series of linear peptoids against mammalian cell lines (HaCaT and HepG2). The cytotoxicity of the peptoid library has then been correlated with their antibacterial properties against Gram-positive and Gram-negative bacteria and also to the hydrophobicity of the peptoid sequences. The work presented provides valuable data to aid in the future rational design of antimicrobial peptoids.

## Abstract 2

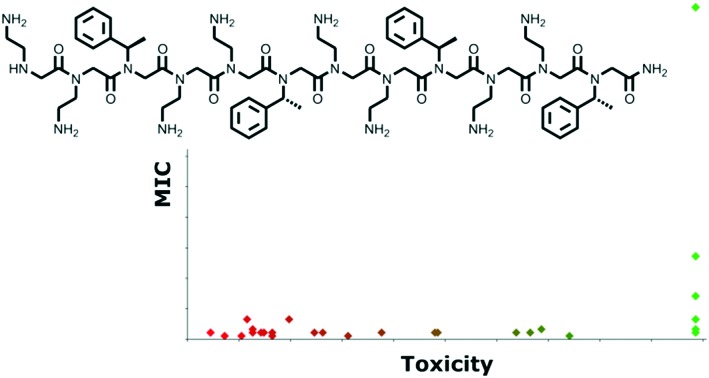
The cytoxicity profiling of a series of linear peptoids against mammalian cell lines has been carried out and correlated against both antibacterial properties and hydrophobicity.

## 

The growing prevalence of antibiotic resistance has intensified demand for novel antimicrobials to replace or complement existing treatments for infectious diseases. Recent incentives such as the 10 × '20 Initiative and the Global Antimicrobial Resistance Research Innovation Fund encourage investment and a commitment to the development of new antibacterial drugs.^1^ Given the effectiveness of the innate immune system in providing the first line of defence against infection, considerable research effort has focused on investigating the activities of antimicrobial peptides (AMPs), with a view to their deployment as templates for innovative therapeutic design.^2^–^5^ AMPs typically contain fewer than 50 amino acids, are cationic and play a fundamental role in host defence, functioning as both antimicrobial agents and modulators of the inflammatory response.^6^–^11^ AMPs display potent antimicrobial activity against a range of clinically important pathogens, including bacterial, fungal and parasitic species.^12^–^16^ However, despite promising biological properties, AMPs are highly susceptible to degradation by host proteinases, which has hindered their exploitation as novel therapeutics including limited success in clinical trials.^2^–^8^,^15^–^19^

In the search for peptidomimetics that retain potent antimicrobial activity yet also exhibit enhanced proteolytic stability, α-peptoids (*N*-substituted glycines) have emerged as highly promising candidates. In α-peptoids, the side chain of each residue (monomer) is located on the nitrogen in the amide backbone (Fig. 1). This allows peptoids to keep many of the advantageous properties of AMPs (*e.g.* amphilicity) but, with the inclusion of a tertiary amide backbone, significantly improve their resistance to enzymatic degradation.^20^

**Fig. 1:**
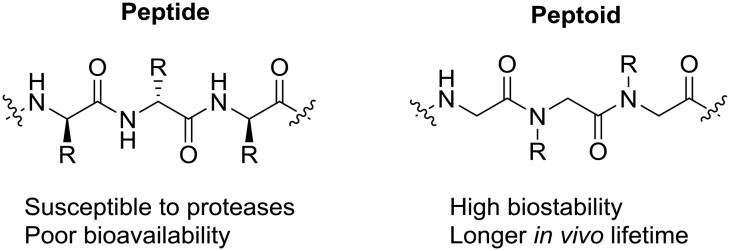
Representative structures of an α-peptide and an α-peptoid.

Peptoids have been shown to have potency against a wide variety of Gram-positive and negative bacteria,^21^–^30^ parasites^31^–^33^ and fungi.^25^,^34^–^36^ These studies highlight the clear potential that peptoids offer as a new class of antimicrobials but in order to progress their clinical development a more detailed study of their toxicity towards mammalian cells needs to be undertaken.

Peptoid toxicity is often evaluated by haemolytic activity, however, these assays cannot necessarily be used to predict toxicity more generally against mammalian cells. For example, many peptoids in the literature are based upon peptoid (*N*Lys*N*spe*N*spe)_4_ (compound **13**, Table 1) which exhibits excellent antimicrobial properties, with a reported selectivity ratio of >6 and acceptable 50% haemolytic dose (HD_50_ of 100 μM).^23^ However, more recently, another publication reports *in vitro* mammalian assays and observed toxicity of peptoid **13** at 5.1 μM against NIH 3T3 murine fibroblasts.^37^ The Olsen group have also demonstrated that α-peptide β-peptoid hybrids show negligible haemolytic activity, but cause severe membrane alterations to human erythrocytes at low concentrations *via* microscopy.^31^ Therefore, there is a clear need to scrutinise the relationship between peptoid antimicrobial activity and their toxicity towards mammalian cells in greater detail.

**Table 1:** Toxicity, antibacterial activity and associated selectivity values for the compounds studied. Selectivity is represented as an average of the selectivity to HaCaT and HepG2 (where selectivity = toxicity/activity). All data and unaveraged selectivity values can be found in the ESI. *ND* – compounds were not tested due to a lack of material, but are included here as some data was collected against certain species and they represent novel peptoid structures. References show publications where the same sequences have previously been evaluated for their antimicrobial activity

Peptoid sequence		Ref.	ED_50_ (μM)		MIC (μM)				Average selectivity			
			HaCaT	HepG2	*E. coli*	*P. aeruginosa*	*S. aureus*	*S. epidermidis*	*E. coli*	*P. aeruginosa*	*S. aureus*	*S. epidermidis*
(*N*ah*N*phe*N*phe)_4_	**1**	33	>100	>100	13	>100	2	6	8	1	50	17
(*N*ah*N*phe*N*phe)_3_	**2**	33	>100	>100	50	>100	6	3	2	1	17	33
(*N*ah*N*phe*N*phe)_2_	**3**	33	>100	>100	>100	>100	>100	>100	1	1	1	1
(*N*Lys*N*phe*N*phe)_4_	**4**	33, 37	36	>100	13	50	3	2	6	2	23	34
(*N*Lys*N*phe*N*phe)_3_	**5**	33	>100	>100	50	>100	25	6	2	1	4	17
(*N*Lys*N*phe*N*phe)_2_	**6**	33	>100	>100	>100	>100	>100	>100	1	1	1	1
(*N*ae*N*phe*N*phe)_4_	**7**	33	>100	>100	13	50	2	6	8	2	50	17
(*N*ae*N*phe*N*phe)_3_	**8**	33	>100	>100	>100	>100	13	>100	1	1	8	1
(*N*ae*N*phe*N*phe)_2_	**9**	33	>100	>100	>100	>100	>100	>100	1	1	1	1
(*N*ah*N*spe*N*spe)_4_	**10**	33	23	41	25	50	2	2	2	1	16	17
(*N*ah*N*spe*N*spe)_3_	**11**	33	>100	>100	25	>100	3	2	4	1	33	50
(*N*ah*N*spe*N*spe)_2_	**12**	33	>100	>100	>100	>100	>100	25	1	1	1	4
(*N*Lys*N*spe*N*spe)_4_	**13**	21, 23, 33	20	29	25	50	2	1	1	1	13	25
(*N*Lys*N*spe*N*spe)_3_	**14**	23, 33	>100	>100	13	>100	2	2	8	1	50	50
(*N*Lys*N*spe*N*spe)_2_	**15**	23, 33	>100	>100	>100	>100	>100	25	1	1	1	4
(*N*ae*N*spe*N*spe)_4_	**16**	33	26	41	>100	50	2	2	0	1	17	17
(*N*ae*N*spe*N*spe)_3_	**17**	33	>100	>100	25	>100	2	13	4	1	50	8
(*N*ae*N*spe*N*spe)_2_	**18**	33	>100	>100	>100	>100	>100	>100	1	1	1	1
(*N*Lys*N*pmb*N*pmb)_4_	**19**		41	>100	>100	>100	3	2	1	1	24	36
(*N*Lys*N*pcb*N*pcb)_4_	**20**		18	22	>100	>100	25	6	0	0	1	4
(*N*Lys*N*pcb*N*pcb)_3_	**21**		22	23	50	25	3	2	0	1	8	12
(*N*Lys*N*pfb*N*pfb)_4_	**22**		46	30	13	25	2	1	3	2	19	38
(*N*Lys*N*pfb*N*pfb)_3_	**23**		>100	45	13	25	3	3	6	3	24	24
(*N*Lys*N*mfb*N*mfb)_4_	**24**		25	17	25	25	6	3	1	1	4	7
(*N*Lys*N*mfb*N*mfb)_3_	**25**		64	43	13	25	6	2	4	3	9	27
(*N*Lys*N*pfb*N*spe)_4_	**26**		20	26	13	13	2	2	2	2	12	12
(*N*Lys*N*pfb*N*spe)_3_	**27**		52	36	25	25	3	2	2	2	15	22
[(*N*Lys*N*pfb*N*pfb)(*N*Lys*N*spe*N*spe)]_2_	**28**		>100	55	6	50	2	1	13	2	39	78
(*N*Lys*N*spe*N*spe)(*N*Lys*N*pfb*N*pfb)(*N*Lys*N*spe*N*spe)	**29**		65	43	13	25	3	2	4	3	21	28
(*N*amy*N*spe*N*spe)[(*N*Lys*N*spe*N*spe)]_3_	**30**		12	15	50	50	2	2	0	0	7	7
(*N*amy*N*spe*N*spe)_2_(*N*Lys*N*spe*N*spe)_2_	**31**		20	18	>100	>100	2	1	0	0	10	19
(*N*Lys*N*spe*N*spe)_2_(*N*amy*N*spe*N*spe)(*N*Lys*N*spe*N*spe)	**32**		20	22	>100	>100	6	2	0	0	4	11
(*N*hArg*N*phe*N*phe)_4_	**33**		>100	*ND*	6	25	*ND*	2	17	4	—	50
(*N*hArg*N*spe*N*spe)_4_	**34**		20	12	6	13	1	1	3	2	16	16
(*N*hArg*N*spe*N*spe)_3_	**35**		*ND*	*ND*	6	50	2	2	—	—	—	—
(*N*hArg*N*mfb*N*mfb)_4_	**36**		28	21	13	13	2	2	2	2	12	12
(*N*hArg*N*mfb*N*mfb)_3_	**37**		*ND*	*ND*	6	25	2	1	—	—	—	—
(*N*hArg*N*hLeu*N*spe)_4_	**38**		*ND*	*ND*	13	25	1	2	—	—	—	—
(*N*hArg*N*hLeu*N*spe)_3_	**39**		*ND*	*ND*	*ND*	>100	3	2	—	—	—	—
[(*N*amy*N*spe*N*spe)(*N*hArg*N*spe*N*spe)]_2_	**40**		31	24	>100	>100	3	6	0	0	9	5
(*N*Lys*N*spe*N*spe)_2_(*N*hArg*N*spe*N*spe)_2_	**41**		>100	*ND*	17	34	17	*ND*	6	3	6	—
(*N*hArg*N*spe*N*spe)_2_(*N*Lys*N*spe*N*spe)_2_	**42**		15	*ND*	17	17	17	*ND*	1	1	1	—
(*N*Lys*N*spe*N*spe)(*N*hArg*N*spe*N*spe)(*N*Lys*N*spe*N*spe)_2_	**43**		33	*ND*	17	17	17	*ND*	2	2	2	—
[(*N*hArg*N*spe*N*spe)(*N*Lys*N*spe*N*spe)]_2_	**44**		33	*ND*	17	67	17	*ND*	2	0	2	—

In this study we report the synthesis of one of the largest single library of antimicrobial peptoids published to date, in order to undertake a structure–activity relationship (SAR) of the wide variety of chemical functionalities present. Some of the sequence motifs and monomers represented here have not yet been reported in any antibacterial peptoids. This study focuses on the antibacterial potency of these peptoids against representative Gram-positive and Gram-negative species and significantly, we also examine the toxicity of this library using therapeutic indices against representative mammalian cell lines to evaluate the potential of peptoids as novel anti-infective compounds.

## Results and discussion

### Library design

We previously described the antiparasitic activities of a small library of linear peptoids.^33^ In this work, 18 peptoids from this first library have been retested to investigate their antibacterial activity and toxicity profiles (peptoids **1–18**, Table 1), which to date have not been reported. An additional 26 novel peptoids were also synthesised to conduct a broader SAR study (peptoids **19–44**, Table 1). This library represents the largest library of anti-infective peptoids published to our knowledge.

Since the presumed mode of action of AMPs is by membrane disruption, the peptoid sequences selected contain a variety of side chain functionalisation to help elucidate the features necessary for activity. A defined secondary structure is thought to promote the antimicrobial action of both peptoids and peptides, therefore all peptoids tested here were designed around a repeating trimer motif where the third monomer was typically a cationic monomer (a peptoid helix turn is reported to occur every three residues in a polyproline type I helix) and often included the α-chiral *N*spe or *N*(*S*-phenylethyl) glycine monomer in order to help induce an amphipathic secondary structure.^23^,^38^

**Fig. 2:**
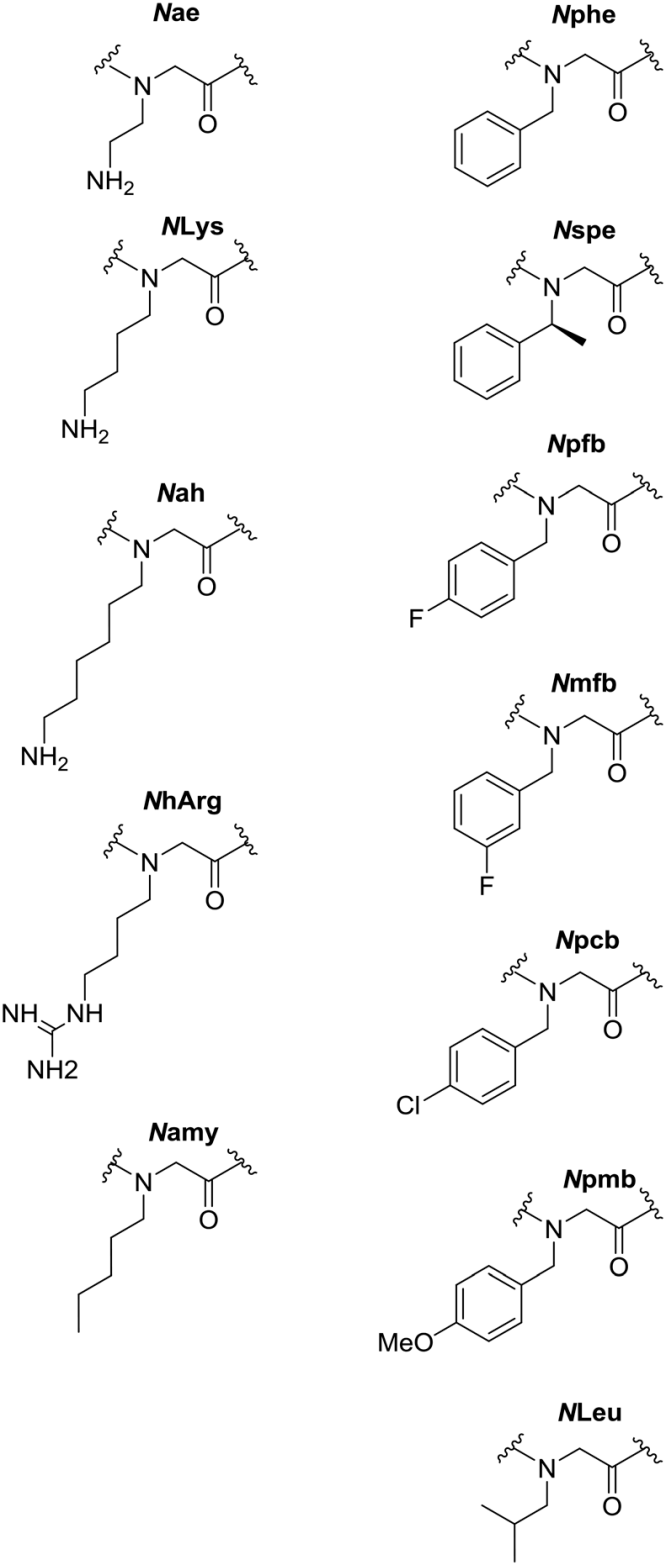
Peptoid monomers utilised in this study.

All peptoids were either synthesised manually on-resin or using an automated synthesiser using the submonomer method of synthesis at room temperature. Compounds between six and twelve residues in length were made, corresponding to peptoids with an overall positive charge of +2, +3 or +4.^39^,^40^ This extended library includes a wide range of monomers, such as those containing alkyl and substituted aromatic residues, including chlorinated and fluorinated peptoids. In addition, we have included peptoids that have different cationic functionalities in the sequence to further probe the relationship between charge and activity (Fig. 2).

Previously we described the activity of peptoids that contain lysine monomers (amino-functionalised cationic residues).^32^,^33^ It has been suggested that arginine-containing peptoids may have increased membrane permeability^41^ and so a small number of arginine analogues of our lysine library were generated. In addition, we recently described the synthesis of peptoids with mixed lysine and arginine functionalities, which are some of the first examples of this class of compound to be reported in the literature.^42^

### Antimicrobial activity

The activity of our peptoid library was assessed against both bacterial and mammalian cells to gain a broader understanding of the factors that lead to effective and selective antimicrobial peptoids. We screened for broad-spectrum antibacterial activity against Gram-negative (*Escherichia coli* and *Pseudomonas aeruginosa*) and Gram-positive (*Staphylococcus aureus* and *S. epidermidis*) bacteria. The toxicity of the library was also assessed against two mammalian cell lines; HaCaT spontaneously transformed aneuploid keratinocytes were employed to model toxicity against human skin cells and HepG2, a cell line derived from a human liver carcinoma, were used as a study on polarised human hepatocytes. Detailed methods for these assays can be found in the ESI.‡

In order to compare the activity of our compounds between the minimum inhibitory concentration (MIC) values obtained for bacteria and the effective dose (ED_50_) measurements against eukaryotic cells, all results have been presented in μM units. As seen from the data summarised in Table 1, many peptoids within the library have low MIC values against both Gram-negative and Gram-positive bacteria, ranging from the most active at less than 1 μM to inactive peptoids with no activity even at 100 μM. Some of the MIC values obtained are within the same range of selected natural antimicrobial peptides described in the literature. For example, the AMP cecropin A was shown to kill 90% of *E. coli* at 2 μM (ref. 43) and peptoids **33**, **34**, **35** and **37** all have an MIC of 6 μM against *E. coli*. Magainin 2, an amphibian derived AMP, was reported to have MICs against *E. coli*, *S. epidermidis* and *S. aureus* of 5 μg mL^–1^ (2 μM), 10 μg mL^–1^ (4 μM) and 50 μg mL^–1^ (20 μM) respectively and many of the peptoids in Table 1 exhibit even better antibacterial activities.^43^,^44^

Unsurprisingly, the activity of most of the peptoids is significantly greater against the Gram-positive species (*S. aureus* and *S. epidermidis*) than the Gram-negative *E. coli* and *P. aeruginosa*. This differential activity is probably due to the presence of the lipopolysaccharide-rich outer membrane of Gram-negatives, which presents a significant permeability barrier to many hydrophobic molecules.^45^,^46^ Certain compounds within the library displayed selectivity for particular bacterial species, For example, compound 17 had an MIC of 25 μM against *E. coli*, but >100 μM against the other Gram-negative bacterium, *P. aeruginosa* or 20 which has an MIC of 6 μM against *S. epidermidis* but only moderate activity of 25 μM against *S. aureus*. However, no Gram-negative specific antibacterial peptoids were identified. Any sequences that can selectively target Gram-negative bacteria are highly sought after due to rising concerns over antibiotic resistance.^47^–^49^

### Structure–activity relationships: simple library

Factors necessary for robust activity against the protozoan intracellular parasite *Leishmania mexicana* were previously determined and include sequence length and inclusion of chiral monomers.^31^ Side chain length of cationic residues was also identified as an important feature for efficacy, with *N*ae or *N*Lys displaying improved antiparasitic activity over the longer *N*ah residue.^33^ The same features are replicated here in the activity of the peptoid library against differing bacterial species. The longest 12 residue peptoids (**1**, **4**, **7**, **10**, **13** and **16**) were always more active than their 9 residue analogues (**2**, **5**, **8**, **11**, **14** and **17**), which were themselves more active than the 6 residue sequences (*i.e.***3**, **6**, **9**, **12**, **15** and **18**), conclusions that agree with those from the Barron group.^23^ Hexapeptoids **3**, **6**, **9**, **12**, **15** and **18** showed generally limited antibacterial activity against the bacteria tested with MICs of 100 μM, although it is interesting to note that *S. epidermidis* did show some increased sensitivity to peptoids **12** and **15** (Table 1).

Interestingly, the effect of monomer chirality was less important than with *L. mexicana* in achieving antibacterial activity; in many cases, sequences comprised exclusively from achiral monomers had comparable or better activity than analogues containing the chiral *N*spe building block. For example, comparing peptoid **4** to **13** which are achiral and chiral analogues of the same sequence, we observe similar MIC values against *P. aeruginosa* (50 μM for both) and *S. aureus* (MIC 3 μM and 2 μM). However, against *E. coli*, the achiral peptoid **4** has an MIC of 12 μM compared to 25 μM for the chiral equivalent **13**. A similar pattern is apparent with peptoids **7** and **16** (sequences with *N*ae and either *N*phe or *N*spe respectively), with the peptoids having similar activity against both *P. aeruginosa* and *S. aureus* but the achiral sequence **7** shows better activity against *E. coli* (MIC 13 μM and >100 μM respectively).

When comparing sequences containing different amino-functionalised cationic monomers, *N*ae, *N*Lys and *N*ah, it is not possible to draw a simple conclusion about the optimum length of side chain for best antibacterial activity since promising activity is evident with all three cationic residues. Peptoids based upon the *N*x*N*phe*N*phe motif **1** (*N*ah), **4** (*N*Lys) and **7** (*N*ae) all have an MIC of 13 μM against *E. coli* and good activity against both *Staphylococcus* species. However, when comparing the chiral analogues of the same motif (*N*spe replaced *N*phe), the sequences with *N*ah and *N*Lys (**10** and **13**) have an MIC of 25 μM against *E. coli* but the peptoid with the shortest *N*ae residue (**16**) has negligible activity. For peptoids **10**, **13** and **16**, the activity against *P. aeruginosa*, *S. aureus* and *S. epidermidis* is similar regardless of the choice of cationic monomer (Table 1).

### Effects of aromatic building block substitution

A selection of substituted aromatic monomers were included in a similar repeating motif of two aromatic monomers followed by the charged *N*Lys residue to determine their impact on biological activity. Monomer substitutions included a methoxy group (*N*pmb), chlorine (*N*pcb) or fluorine in both *para* and *meta* positions (*N*pfb, *N*mfb). The effect of these substitutions on anti-Leishmanial activity were reported recently.^32^ It was shown that halogenated monomers (in particular fluorinated ones) improve the efficacy of peptoid sequences against amastigotes. Based upon the success of this approach, we concentrated our efforts on the antibacterial screening of halogenated sequences.

Methoxy substituted peptoids were only tested at the longest 12 residue length (peptoid **19**) and show a negligible effect against the two Gram-negative species and no improvement in action against the Gram-positive bacteria compared to the unsubstituted analogue (peptoid **4**). Addition of chlorine in the *para* position improves activity against *L. mexicana* axenic amastigotes, however decreases antibacterial activity (*i.e.* compounds **20***cf.***4** with an MIC of >100 μM and 13 μM with *E. coli*) and also significantly increases the toxicity of the sequences to mammalian cells (ED_50_ HepG2 22 μM and >100 μM respectively).

Fluorinated peptoid sequences were more successful at targeting the various bacteria. Peptoids with exclusively achiral monomers (*N*pfb or *N*mfb) and those with a mixture of chiral and achiral building blocks (*N*pfb and *N*spe) were tested. Those sequences containing the achiral monomers (peptoids **22–25**) have marginally improved antibacterial activities compared to analogues of the same length with *N*phe rather than fluorinated monomers (peptoids **4–6**). There seems to be no significant difference between monomers substituted in the *para* or *meta* position. Interestingly, the 9-residue peptoids **23** and **25** have a similar level of broad spectrum antibacterial activity as those with 12 residues (**22** and **24**), but the former show reduced toxicity to mammalian cells. The 6-residue sequences display reduced activity against bacteria. In this case, it appears that the shorter 9-residue sequences **23** and **25** may prove to be better antibacterial candidates with a larger therapeutic window between activity and toxicity (Table 1)

The simple chiral sequences (*i.e.***13**) are more potent, but also more toxic than the achiral *N*phe peptoids (**4–6**). Given that the fluorinated, achiral monomers exhibit an increase in antibacterial activity compared to the unsubstituted analogues, the *N*pfb monomer was also placed into the following motif: *N*Lys*N*pfb*N*spe to examine whether the activity of chiral sequences could be modulated (peptoids **26** and **27**). Further iterations were also synthesised where the *N*pfb and *N*spe monomers were placed in a coblock manner (*i.e.* sequences **28** and **29**). In both templates, the longest sequences showed the best broad-spectrum antibacterial activity. In particular, the 12-residue block peptoid (**28**) is promising, with reduced toxicity compared to sequences made of *N*spe or *N*pfb exclusively and improved activity against *E. coli* (MIC 6 μM), *S. aureus* and *S. epidermidis* (MICs 2 μM and <1 μM).

### Peptoids containing alkyl chains

To probe the relationship between net charge and hydrophobicity of a peptoid and its biological activity, analogues of peptoid **13** were synthesised where the cationic *N*Lys monomer was substituted by the alkyl monomer *N*amy. By replacing the charged amino group with a methyl group, the overall compound charge is reduced, however the number of atoms in the side chain and overall molecular weight remains unaltered. In these analogues (peptoids **30–32**), the charge is replaced at just the *N* terminal end of the sequence or at two positions within the sequence.

When the antibacterial properties are considered, there is little difference in efficacy against the Gram-positive bacteria tested, however, the substitutions lead to a reduction in overall activity against Gram-negatives. The parent sequence **13** shows moderate activity against *E. coli* and *P. aeruginosa* (MICs of 25 μM and 50 μM respectively) and potent micromolar activity against the Gram-positives. However, peptoids **30–32** have MICs of 50 μM or >100 μM against *E. coli* and similar reductions in activity are seen for *P. aeuriginosa*.

### Substitution of arginine-instead of lysine-residues

As described in the literature,^31^,^41^,^50^ arginine-containing peptoids are known to increase membrane permeability and antibacterial activity. Hence, sequences containing arginine peptoid monomers were included in the library. This allowed the comparison of differently functionalised cationic residues on the peptoid sequences (*i.e.* the amino *N*Lys peptoids or the guanidine groups of *N*hArg). It has also been suggested that arginine in peptide sequences can improve antibacterial potency, although this is also linked with enhanced toxicity.^26^,^31^ In an attempt to modulate the biological properties of the peptoid library, for the first time, peptoids with both lysine and arginine in the same sequence were evaluated against bacterial targets using the new methodology developed in our group.^42^

This sublibrary of peptoids can be split between sequences exclusively containing arginine residues (peptoids **33–40**) and those that contain a mixture of both lysine and arginine-type side chains (peptoids **41–44**). In these compounds *N*hArg was introduced, which is the equivalent side chain to the *N*Lys residue, with 4 carbons in the backbone and the terminal guanidine moiety.

In contrast to peptoids that contain amino-functionalised *N*Lys residues the *N*hArg sequences tend to have an increased toxicity to the mammalian cell lines tested but do also display improved activity against the bacteria tested. For example, when comparing the fluorinated peptoids **36** and **37** to their lysine-equivalents (**24** and **25** respectively) we see an approximate 2-fold increase in antibacterial activity for all species tested; for the longest 12-residue sequence (*N*x*N*mfb*N*mfb)_4_ activity against *E. coli* is 13 μM in **36** with *N*hArg, compared to 25 μM in **24** (*N*Lys). Against *S. aureus* the arginine-type peptoid has an MIC of 2 μM contrasting with 6 μM for the lysine equivalent. However, in sequences that were inactive with lysine residues, replacement by *N*hArg does not make the sequence active (see peptoid **40**, where the sequence is not active against Gram-negative bacteria at any concentration), but in these cases the inclusion of the guanidine group does increase the toxicity.

As predicted, sequences with a combination of lysine and arginine-type residues show a balance between toxicity to mammalian cells and antibacterial activity compared to sequences containing *N*Lys or *N*hArg residues exclusively. For example, in related peptoids containing all arginine residues (**34**), all lysine residues (**13**) and both lysine/arginine monomers within the same sequence (peptoids **42–44**), we see toxicity to the HaCaT keratinocytes at 11 μM, 20 μM and 15–33 μM respectively. The general antibacterial activity follows a similar trend, for example, against *E. coli* the lysine-only peptoid **13** has the lowest activity at 25 μM, the arginine-only peptoid **34** has the most potent activity with an MIC of 6 μM and the mixed sequences **42–44** have intermediate activity at 17 μM.

The observation that guanidine-only peptoids display the most potent biological activities is in agreement with previous studies into arginine-rich peptides, which are able to bind membrane-bound lipids more readily than their amino-functionalised lysine equivalents.^31^,^51^ In this case it was proposed that the arginine-type side chains can form bidentate hydrogen bonds with the phospholipid head groups. This conclusion was also reached in studies with antimicrobial peptide–peptoid hybrids containing both lysine and arginine.^31^,^51^

### Toxicity

From the antibacterial MIC determination in Table 1, multiple promising peptoids were identified that showed little or no toxicity to either of the mammalian cell lines tested. For example, compounds **4**, **7**, **23** and **28** display negligible toxicity to HaCaT or HepG2 at the highest concentrations used and are also broad spectrum antibacterial agents. However, many of the sequences generated did display significant toxicity to mammalian cells, and in general these compounds were similarly toxic to both HaCaT and HepG2. On the whole, as the antimicrobial action of a compound increases, the associated toxicity is also increased. This is a problem found in other recent studies that focused on the biological applications of peptoids, however, attention is frequently not directed towards the issue of toxicity.^26^,^29^,^37^

**Fig. 3:**
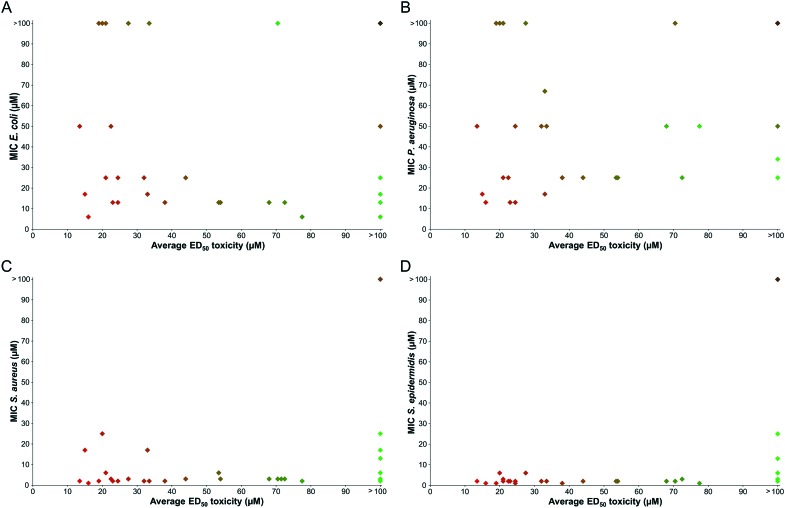
Antibacterial activity of peptoids plotted against the average toxicity to mammalian cell lines (HaCaT and HepG2). A: *E. coli*, B: *P. aeruginosa*, C: *S. aureus*, D: *S. epidermidis*. Compounds indicated in green show activity against prokaryotes with reduced toxicity to eukaryotes, those in red show increased toxicity to mammalian cells and/or weaker activity against the bacteria.

The selectivity of sequences is highlighted as a particular challenge for the design of antimicrobial peptoids. To explore the relationship between activity and toxicity a comparison was made between the MIC values against each bacterial species *versus* the average toxicity of each peptoid to HaCaT and HepG2 (see Fig. 3).

A handful of peptoids were identified that show respectable antibacterial activity and also display low toxicity to mammalian cells. These compounds have the potential to be future selective antibiotic compounds. However, a large proportion of the peptoids within this library do display significant toxicity. There are also many sequences that are toxic to mammalian cells, but show little activity against Gram-negative bacteria. It is likely that the external lipopolysaccharide layer on the outer membrane of both *E. coli* and *P. aeruginosa* (absent in Gram positive bacteria) prevents these peptoids from reaching the cell membrane. To investigate a possible explanation for this observation, the hydrophobicity of the compounds was considered.

### Hydrophobicity

**Fig. 4:**
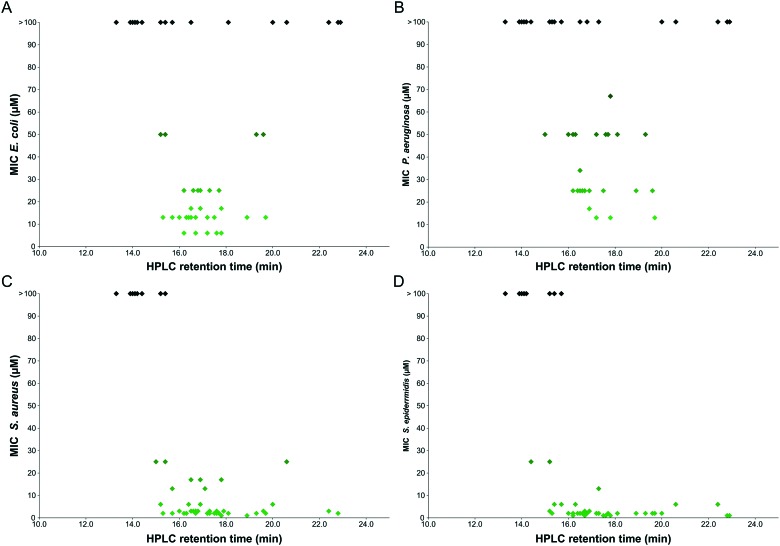
Antibacterial activity of peptoids plotted against HPLC retention times. A: *E. coli*, B: *P. aeruginosa*, C: *S. aureus*, D: *S. epidermidis*. The HPLC gradient from 0–100% B was conducted over 30 minutes with a column oven at 40 °C, where solvent A was 95% H_2_O, 5% MeCN, 0.1% TFA and solvent B was 95% MeCN, 5% H_2_O, 0.1% TFA. Peptoids, shown in green, show antibacterial activity, those in black have negligible activity.

The hydrophobicity of our library was assessed using reverse-phase HPLC retention times as in Fig. 4. Although this provides only a relatively crude measure of hydrophobicity and may not translate directly into predictions about how a compound will interact with the cell membranes of biological systems, certain interesting trends were evident from the analysis.

Since the activity of peptoids is much greater against Gram-positive bacteria than Gram-negative bacteria, the graphs are predominantly populated by peptoids for *E. coli* and *P. aeruginosa* due to their higher MIC values. There appears to be a linear relationship between activity and retention time for the Gram-positive bacteria, where the peptoids at longer retention times (therefore more hydrophobic) have the lowest MIC values against *S. aureus* or *S. epidermidis*.

However, for the Gram-negative bacteria, there is no clear correlation between hydrophobicity and antibacterial activity of the peptoid library. Some compounds with the longest retention times (presumably the most hydrophobic) are inactive against these bacteria, whereas others with shorter retention times display good activity. It has previously been suggested that highly hydrophobic sequences may have lower activities due to self-association, preventing sufficient contact with the cell membrane.^29^ However, this may be an oversimplification since the same results are not observed with Gram-positive bacteria, where the same hydrophobic peptoid sequences result in low MIC values.

The discovery that peptoids with high hydrophobicity are not always the most potent against *E. coli* and *P. aeruginosa*, but against *S. aureus* a linear relationship is seen between hydrophobicity and activity is corroborated by a recent report.^29^ However, the lack of a consensus between hydrophobicity and activity in published libraries, highlights the need for the research community to develop additional tools to help predict peptoid properties and likely efficacy.

**Fig. 5:**
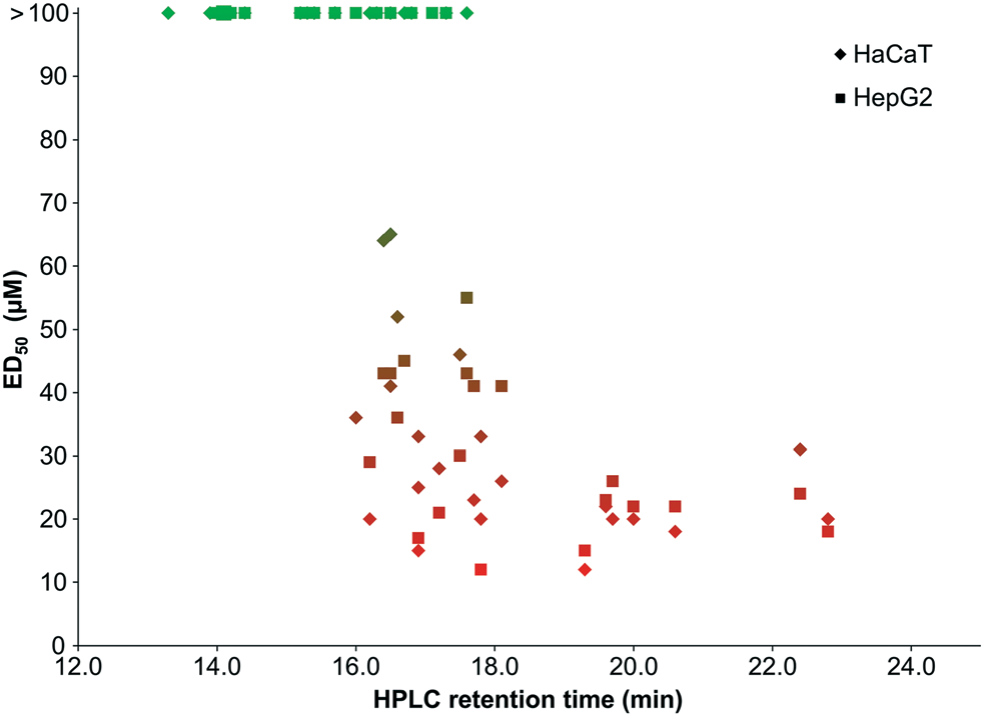
Peptoid toxicity plotted against HPLC retention time. HPLC gradient from 0–100% B over 30 minutes, column oven at 40 °C, where solvent A is 95% H_2_O, 5% MeCN, 0.1% TFA and solvent B is 95% MeCN, 5% H_2_O, 0.1% TFA. Compounds indicated by green show negligible toxicity, those in red show toxicity to mammalian cells.

There also seems to be a correlation between the toxicity of our peptoid library to mammalian cells and compound hydrophobicity, with a similar profile of toxicity for both HaCaT and HepG2 (see Fig. 5). The least hydrophobic compounds are generally the least toxic, whereas those with the highest retention times show the lowest ED_50_ values.

In the literature, HPLC retention times are used to rationalise antimicrobial activity^21^,^23^,^29^ and also for *in silico* predictions of activity.^37^ From the data presented here, it is suggested that HPLC retention time alone cannot estimate or rationalise activity. For Gram-negative bacteria, HPLC retention time is not predictive of activity and although peptoids with longer retention times may have an increased efficacy against Gram-positive bacteria, these compounds may also have a concomitant and undesirable increased toxicity.

Other parameters to evaluate the activity of antimicrobial peptoids and guide the design of bioactive peptoids need to be considered. We recently proposed hydrophobicity measurements determined *via* partitioning experiments (*i.e.* log *D*) as an improved approach to rationalise the biological activity of peptoids.^52^

## Conclusions

This study presents a large and varied library of peptoids that were specifically designed to mimic natural antimicrobial peptides. Many of the peptoids generated show potential as antimicrobial compounds, with broad-spectrum activity against a wide variety of bacterial and parasitic targets and may be promising leads in the future for antibiotics that can combat the increasing problem of resistance.

Most of the peptoids were considerably more active towards Gram positive species than Gram-negatives in keeping with their differing envelope structures. Hence the peptoid library has much better therapeutic indices against *S. aureus* and *S. epidermidis*. The best activity against *E. coli* reported was 6 μM for peptoids **28**, **33**, **34**, **35** and **37** and 13 μM against *P. aeruginosa* for peptoids **26**, **34** and **36**. Many peptoids also showed micromolar activities against *S. aureus* and *S. epidermidis* with both species showing a similar pattern of sensitivity; *S. aureus* did show a tendency to be more resistant than *S. epidermidis* possibly due to minor differences in cell surface charge and hydrophobicity.^53^,^54^ In many cases, *P. aeruginosa* also proved more peptoid-resistant than *E. coli*. This difference may reflect variation in lipid composition or the capacity of *P. aeruginosa* to form biofilms and has been observed previously with AMPs, such as LL-37.^55^

Factors contributing to enhanced antimicrobial activity include the overall length of sequence, with longer, 12-residue peptoids typically displaying the best activity, although in many cases the 9-residue peptoids also display broad spectrum activity against bacteria. Substitution of fluorine in peptoid monomers also enhances activity and in many cases, achiral sequences displayed potency, especially against the Gram-positive species. Interestingly, from our library most compounds that are active against the bacteria but exhibit no/low toxicity to mammalian cells contain achiral monomers, or at least a reduced frequency of chiral monomers within the sequence compared to the standard *N*x*N*spe*N*spe motif. Whether through the presumed increase in hydrophobicity, or through other effects, the addition of chiral *N*spe residues in a sequence often has a detrimental effect on overall toxicity. Since many of the achiral sequences show potent antibacterial activity and these peptoids are not expected to form fully folded peptoid helices, secondary structure is perhaps not as important as predicted.

It is generally stated that the mode of action for AMPs/peptoids is cell membrane disruption.^47^,^56^,^57^ For certain compounds, particularly the more hydrophobic compounds that are both active and toxic, this may be the case. However, the differences exhibited by some compounds, particularly those with negligible toxicity to mammalian cells yet good activity against bacteria, may indicate that cell membrane disruption is not the only mechanism at work.^46^ Current work within our group is now focussing on investigating the molecular mechanism of a selection of these compounds to elucidate the factors necessary for antimicrobial selectivity and potency.

The peptoid community clearly has the requisite tools to design and synthesise effective antimicrobial peptoid sequences with potential for clinical application, however, the challenge now is to focus on increasing pathogen selectivity while minimising host cell toxicity.

### Data availability

In addition to the material provided in the ESI underlying research data for this paper is also available in accordance with the EPSRC open data policy from doi: 10.15128/r1zg64tk92g.

## Experimental

### Materials and reagents

Abbreviations for reagents are as follows: *tert*-butoxycarbonyl (Boc); 9-fluorenylmethoxylcarbonyl (Fmoc); trifluoroacetic acid (TFA); triisopropylsilyl (TIPS); *N*,*N*-dimethylformamide (DMF); *N*,*N*-diisopropylcarbodiimide (DIC); dimethylsulphoxide (DMSO). Solvents and reagents were purchased from commercial sources and used without further purification unless otherwise stated. Rink amide resin (typical loading level 0.6–0.8 mmol g^–1^) was purchased from Merck4Biosciences. DMF was purchased from AGTC Bioproducts (National Diagnostics). Piperidine, bromoacetic acid and TFA were purchased from Sigma Aldrich. The amine building blocks were sourced from Sigma Aldrich or TCI Europe.

### Peptoid synthesis procedures

Peptoids in this library were synthesised both manually and on an automated synthesiser. Protocols for each synthesis method follow.

### Manual linear peptoid synthesis

Fmoc-protected Rink Amide resin (normally 100 mg, 0.1 mmol, typical loading between 0.6–0.8 mmol g^–1^) was swollen in DMF (at least 1 h at room temperature, overnight preferred) in a 20 mL polypropylene Bond Elut SPPS cartridge fitted with two polyethylene frits (Crawford Scientific). The resin was deprotected with piperidine (20% in DMF v/v, 2 × 20 min) and washed with DMF (3 × 2 mL). The resin was treated with bromoacetic acid (1 mL, 0.6 M in DMF) and DIC (0.2 mL, 50% v/v in DMF) for 20 min at room temperature on a shaker platform at 400 rpm (Radleys Technology). The resin was washed with DMF (3 × 2 mL), before the desired amine sub-monomer was added (1 mL, 1.5 M in DMF) and allowed to react for 60 min on the shaker. The resin was again washed with DMF (3 × 2 mL) and the bromoacetylation and amine displacement steps were repeated until the final sub-monomer had been added and the desired peptoid sequence obtained. Resin was washed with DCM and the final cleavage from resin was achieved using a TFA cleavage cocktail (4 ml; TFA : TIPS : H_2_O, 95 : 2.5 : 2.5) on the shaker at 400 rpm for 60 min. The resin was removed by filtration and the cleavage cocktail removed *in vacuo*. The crude product was precipitated in diethyl ether (30 mL) and the precipitate retrieved by centrifuge for 15 min at 5000 rpm. The ether phase was decanted and the crude product dissolved in a mixture of acidified H_2_O and MeCN and lyophilised to a powder before purification.

### Automated linear peptoid synthesis

Automated peptoid synthesis using an Aapptec Apex 396 synthesiser. Fmoc-protected Rink Amide resin (0.1 mmol, loading 0.54 mmol g^–1^) was swollen in DMF (2 mL, 2 min, 475 rpm at RT) and deprotected with 4-methylpiperidine (20% in DMF v/v, 1 mL for 1 min, 475 rpm at RT; then 2 mL for 12 min, 475 rpm at RT). The resin was treated with haloacetic acid solution (either bromo- or chloroacetic acid, 1 mL, 0.6 M in DMF) and DIC (0.18 mL, 50% v/v in DMF) for 20 min at 475 rpm, RT. The resin was washed with DMF (2 mL DMF for 1 min at 475 rpm, ×5) before the desired amine sub-monomer was added (1 mL, 1.5 M in DMF) and shaken for 60 min at 475 rpm. The resin was washed again with DMF (2 mL DMF for 1 min at 475 rpm, ×5) and the acetylation and amine displacement steps were repeated until the desired sequence was achieved. The resin was shrunk in diethyl ether and peptoids cleaved off the resin using a TFA cleavage cocktail (4 ml; TFA : TIPS : H_2_O, 95 : 2.5 : 2.5) for 30–60 min on an orbital shaker at 250 rpm, RT. The cocktail was filtered from the resin and evaporated *in vacuuo* and the resulting residue precipitated in diethyl ether (∼20 ml). The crude peptoid was obtained *via* centrifugation (15 min, 4000 rpm, 5 °C) and the ether layer decanted to yield the crude product as a powder. Peptoids were lyophilised before purification by semi-preparative RP-HPLC.

### Addition of *N*hArg and *N*nArg residues to sequence

To introduce arginine-type residues during the submonomer procedure, the appropriate unprotected diamine was added under normal submonomer coupling conditions (1.5 M amine in DMF, 60 min, room temperature) in place of the mono *N*-Boc diamine and the resin washed with DMF (3 × 2 mL). Dde-OH (10 eq. wrt resin in the minimum volume of DMF) was added to the resin and placed on the shaker at RT for 60 min and the resin washed well with DMF (3 × 2 mL). Subsequent peptoid couplings were made as normal until the desired sequence was achieved, including any extra Dde-protected residues.

After synthesis of the linear peptoid sequence, on resin deprotection of the Dde group was undertaken using 2% hydrazine in DMF (4 × 4 ml × 3 min) and the resin washed with DMF (3 × 2 mL). Guanidinylation of the free amines was achieved using pyrazole-1-carboxamide (6 eq. per free amine, in the minimum amount of DMF) and DIPEA (6 eq. per free amine) on the shaker at 400 rpm, RT for 60 min. The resin was washed with DCM (3 × 2 mL) and shrunk in ether prior to cleavage from the resin, as above.

### Purification by preparative RP-HPLC

Preparative RP-HPLC was performed with a semi-preparative Perkin Elmer Series 200 lc pump fitted with a 785A UV/vis detector using a SB-Analytical ODH-S optimal column (250 × 10 mm, 5 μm); flow rate 2 ml min^–1^; *λ* = 250 nm, where a linear gradient from solvent A to B applied (*A* = 0.1% TFA in 95% H_2_O and 5% MeCN, *B* = 0.1% TFA in 5% H_2_O and 95% MeCN).

### Characterisation

Peptoids were characterised by accurate LC-MS (QToF mass spectrometer and an Acquity UPLC from Waters Ltd) using an Acquity UPLC BEH C8 1.7 μm (2.1 mm × 50 mm) column with a flow rate of 0.6 ml min^–1^ and a linear gradient of 5–95% of solvent B over 3.8 min (*A* = 0.1% formic acid in H_2_O, *B* = 0.1% formic acid in MeCN). Peptide identities were also confirmed by MALDI-TOF mass spectra analysis (Autoflex II ToF/ToF mass spectrometer Bruker Daltonik GmBH) operating in positive ion mode using an α-cyano-4-hydroxycinnamic acid (CHCA) matrix. Data processing was done with MestReNova version 8.1.

Analytical RP-HPLC was carried out using a Perkin Elmer Series 200 lc pump fitted with a series 200 UV/vis detector and autosampler using a SB-Analytical ODH-S optimal column (100 × 1.6 mm, 3.5 μm); flow rate 1 ml min^–1^; *λ* = 220 nm, linear gradient elution 0–100% of solvent B over 30 min (*A* = 0.05% TFA, 95% H_2_O, 5% MeCN, *B* = 0.03% TFA, 5% H_2_O, 95% MeCN).

## Biological assays

### Antibacterial MIC determination

*Please note* – ED_50_, the median effective dose, was defined as the dose that kills 50% of cells.

*Escherichia coli* K-12 wild-type strain (W3110/ATCC27325, F^–^, λ^–^, *rpoS(Am)*, *rph-1*, *Inv*(*rrnD-rrnE*)), *Pseudomonas aeruginosa* PA01 (ATCC 15692) *Staphylococcus aureus* (3R7089 strain Oxford/ATCC9144) and *Staphylococcus epidermidis* (laboratory strain from clinical isolate) were selected for bacteriological studies as representative Gram-negative (*E. coli* and *P. aeruginosa*) and Gram-positive (*S. aureus* and *S. epidermidis*) species. Bacterial cultures were prepared by streaking bacterial strains onto LB agar plates with an inoculation loop and incubated overnight at 37 °C. A single colony was selected and placed in 5 mL of Iso-sensitest broth (Oxoid, ThermoScientific) and incubated with shaking for 16–18 h at 37 °C to provide liquid cultures for testing.

MIC values were obtained according to the protocol described by J. M. Andrews *et al.*^58^ and were conducted in 96-well plates (Sarstedt). Bacteria were grown from overnight cultures in Iso-sensitest broth to an *A*_650nm_ of 0.07 equivalent to a 0.5 MacFarland standard (240 μM BaCl_2_ in 0.18 M H_2_SO_4_). This culture was diluted ten-fold with Iso-sensitest broth before use. Peptoids were initially dissolved in DMSO (5 mM) and diluted further in Iso-sensitest broth to achieve a concentration range of 4–200 μM using 2-fold serial dilutions. 50 μl of inoculum and 50 μl of peptoid solution were added to each test well (final concentration range of 2–100 μM). Experiments were performed in triplicate. A positive control for bacterial growth contained only the inoculum and Iso-sensitest broth. Other controls contained the inoculum and serial dilutions of ampicillin (from 250 μg mL^–1^ to 2 μg mL^–1^), serial dilutions of DMSO and the inoculum to confirm no inhibitory effect on bacterial growth, and Iso-sensitest broth alone as a sterile control. The MIC was defined as the lowest concentration which completely inhibited bacterial growth after incubation at 37 °C for 16 h with shaking. Quantitative data was attained from absorbance values using a Biotek Synergy H4 plate reader.

### Cytotoxicity assay with HepG2 or HaCaT

Cytotoxicity analyses were performed in 96-well plates (Costar, Fisher Scientific) using alamarBlue® (Invitrogen) for cell viability detection using a modified protocol as described previously. HepG2 or HaCaT cells were subcultured at 37 °C, 5% CO_2_ in DMEM high glucose supplemented with heat-inactivated foetal bovine sera (FBS, 10%; Biosera Ltd) and penicillin/streptomycin (P/S, 1%). Cells were counted using a Neubauer Improved Haemocytometer. HepG2 cells were seeded 1 day prior to treatment in 96 well plates at a concentration of 2 × 10^5^ cells per mL in 100 μL of medium (2 × 10^4^ cells per well). Then cells were pre-incubated with the compounds in triplicate in a dilution series in triplicate from 2–100 μM (5 mM stock solutions in DMSO diluted from 100 μM to 3 μM; untreated cells with DMSO as a negative control) in 50 μl of the media for 1 hour. Afterwards, 40 μL were removed from each well before the addition of 90 μL of the media, followed by incubation for 24 hours at 37 °C, 5% CO_2_. Then, 10 μL of alamarBlue® (Invitrogen) was added to each well before a 2 hour incubation prior to assessing cell viability using a fluorescent plate reader (Biotek; Ex 560 nm/Em 600 nm). All data were measured in triplicate on a minimum of two occasions to ensure a robust data set was collected. The ED_50_ values were calculated from the dose response results achieved from the serial dilutions.

## Supplementary Material

Supplementary information
