# Supplementary material for: Exploring the links between peptoid antibacterial activity and toxicity
Source: Medchemcomm. 2017 Feb 1;8(5):886–96. doi: 10.1039/c6md00648e (PMC6072100; doi:10.1039/c6md00648e)
Supplement: Supplementary file 1 [file MD-008-C6MD00648E-s001.pdf]

Electronic Supplementary Material (ESI) for MedChemComm.
This journal is © The Royal Society of Chemistry 2018

Supporting information for

Exploring the links between peptoid antibacterial activity and toxicity

H.L. Bolt[a], G.A. Eggimann[a], C.A.B. Jahoda[b], R.N. Zuckermann[d], G.J. Sharples*[,a,b] and S.L. Cobb*[,a]

[a] Biophysical Sciences Institute, Department of Chemistry, Durham University, South Road, Durham, DH1 3LE, UK.

[b] School of Biological and Biomedical Sciences, Durham University, Durham DH1 3LE, UK.

[c] School of Medicine, Pharmacy and Health, Durham University, Queen's Campus, Stockton-on-Tees, TS17 6BH, UK.

[d] Molecular Foundry, Lawrence Berkeley National Laboratory, Berkeley, California, USA

* Corresponding author, e-mail: s.l.cobb@durham.ac.uk, gary.sharples@durham.ac.uk

Contents

Contents 2

1. Synthetic Procedures 3

   Materials and Reagents 3

   Peptoid Synthesis Procedures 3

   Purification by preparative RP-HPLC 5

   Characterisation 5

2. Biological Assays 6

   Antibacterial MIC determination 6

   Cytotoxicity assay with HepG2 7

   Skin Cell Toxicity assay with HaCaT 7

3. Biological Data 9

4. Characterisation 11

   Characterisation of building blocks and peptoids used in this study 11

   Accurate Mass and Analytical RP-HPLC Data 13

   Analytical HPLC Traces 16

5. References 38

1. Synthetic Procedures

Materials and Reagents

Abbreviations for reagents are as follows: *tert*-butoxycarbonyl (Boc); 9-fluorenylmethoxylcarbonyl (Fmoc); trifluoroacetic acid (TFA); triisopropylsilyl (TIPS); *N,N*-dimethylformamide (DMF); *N,N*-diisopropylcarbodiimide (DIC); dimethylsulphoxide (DMSO). Solvents and reagents were purchased from commercial sources and used without further purification unless otherwise noted. Rink amide resin (typical loading level 0.6-0.8 mmol g^{-1}) was purchased from Merck4Biosciences. DMF was purchased from AGTC Bioproducts (National Diagnostics). Piperidine, bromoacetic acid and TFA were purchased from Sigma Aldrich. The amine building blocks were sourced from Sigma Aldrich or TCI Europe.

Peptoid Synthesis Procedures

Peptoids in this library were synthesised both manually and on an automated synthesiser. Protocols for each synthesis method follow.

Manual Linear Peptoid Synthesis

Fmoc-protected Rink Amide resin (normally 100 mg, 0.1 mmol, typical loading between 0.6–0.8 mmol g^{-1}) was swollen in DMF (at least 1 hour at room temperature, overnight preferred) in a 20 mL polypropylene Bond Elut SPPS cartridge fitted with two polyethylene frits (Crawford Scientific). The resin was deprotected with piperidine (20% in DMF v/v, 2 x 20 min) and washed with DMF (3 x 2mL). The resin was treated with bromoacetic acid (1mL, 0.6M in DMF) and DIC (0.2 mL, 50% *v*/*v* in DMF) for 20 minutes at room temperature on a shaker platform at 400 rpm (Radleys Technology). The resin was washed with DMF (3 x 2 mL), before the desired amine sub-monomer was added (1 mL, 1.5M in DMF) and allowed to react for 60 minutes on the shaker. The resin was again washed with DMF (3 x 2 mL) and the bromoacetylation and amine displacement steps were repeated until the final sub-monomer had been added and the desired peptoid sequence had been obtained. Resin was washed with DCM and the final cleavage from resin was achieved using a TFA cleavage cocktail (4 ml; TFA:TIPS:H_2O, 95:2.5:2.5) on the shaker at 400 rpm for 60 minutes. The resin was removed by filtration and the cleavage cocktail removed *in vacuuo*. The crude product was precipitated in diethyl ether (30 mL) and the precipitate retrieved by centrifuge for 15 min at 5,000 rpm. The ether phase was decanted and the crude product dissolved in a mixture of acidified H_2O and MeCN and lyophilised to a powder before purification.

Automated Linear Peptoid Synthesis

Automated peptoid synthesis using an Aapptec Apex 396 synthesiser. Fmoc-protected Rink Amide resin (0.1 mmol, loading 0.54 mmol g^{-1}) was swollen in DMF (2 mL, 2 min, 475 rpm at RT) and deprotected with 4-methylpiperidine (20% in DMF *v*/*v*, 1 mL for 1 min, 475 rpm at RT; then 2 mL for 12 min, 475 rpm at RT). The resin was treated with haloacetic acid solution (either bromo- or chloroacetic acid, 1 mL, 0.6M in DMF) and DIC (0.18 mL, 50% v/v in DMF) for 20 min at 475 rpm, RT. The resin was washed with DMF (2 mL DMF for 1 min at 475 rpm, x 5) before the desired amine sub-monomer was added (1 mL, 1.5M in DMF) and shaken for 60 mins at 475 rpm. The resin was washed again with DMF (2 mL DMF for 1 min at 475rpm, x 5) and the acetylation and amine displacement steps were repeated until the desired sequence was achieved. The resin was shrunk in diethyl ether and peptoids cleaved off the resin using a TFA cleavage cocktail (4 ml; TFA:TIPS:H_2O, 95:2.5:2.5) for 30-60 min on an orbital shaker at 250 rpm, RT. The cocktail was filtered from the resin and evaporated *in vacuuo* and the resulting residue precipitated in diethyl ether (~20 ml). The crude peptoid was obtained via centrifugation (15 mins, 4,000 rpm, 5 ˚C) and the ether layer decanted to yield the crude product as a powder. Peptoids were lyophilised before purification by semi-preparative RP-HPLC.

Addition of *N*hArg and *N*nArg residues to sequence

To introduce arginine-type residues during the submonomer procedure, the appropriate unprotected diamine was added under normal submonomer coupling conditions (1.5M amine in DMF, 60 minutes, room temperature) in place of the mono *N*-Boc diamine and the resin washed with DMF (3 x 2mL). Dde-OH (10 eq. wrt resin in the minimum volume of DMF) was added to the resin and placed on the shaker at RT for 60 minutes and the resin washed well with DMF (3 x 2mL). Subsequent peptoid couplings were made as normal until the desired sequence was achieved, including any extra Dde-protected residues.

After synthesis of the linear peptoid sequence, on resin deprotection of the Dde group was undertaken using 2% hydrazine in DMF (4 x 4ml x 3 mins) and the resin washed with DMF (3 x 2 mL). Guanidinylation of the free amines was achieved using pyrazole-1-carboxamide (6 eq. per free amine, in the minimum amount of DMF) and DIPEA (6 eq. per free amine) on the shaker at 400 rpm, RT for 60 minutes. The resin was washed with DCM (3 x 2 mL) and shrunk in ether prior to cleavage from the resin, as above.

Purification by preparative RP-HPLC

Preparative RP-HPLC was performed with a semi-preparative Perkin Elmer Series 200 lc pump fitted with a 785A UV/Vis detector using a SB-Analytical ODH-S optimal column (250 × 10 mm, 5 μm); flow rate 2 ml min^{-1}; λ = 250 nm, where a linear gradient from solvent A to B applied (*A* = 0.1% TFA in 95% H_2O and 5% MeCN, *B* = 0.1% TFA in 5% H_2O and 95% MeCN).

Characterisation

Peptoids were characterised by accurate LC-MS (QToF mass spectrometer and an Acquity UPLC from Waters Ltd.) using an Acquity UPLC BEH C8 1.7μm (2.1mm × 50mm) column with a flow rate of 0.6 ml min^{-1} and a linear gradient of 5-95% of solvent B over 3.8 min (*A* = 0.1% formic acid in H_2O, *B* = 0.1% formic acid in MeCN). Peptide identities were also confirmed by MALDI-TOF mass spectra analysis (Autoflex II ToF/ToF mass spectrometer Bruker Daltonik GmBH) operating in positive ion mode using an α-cyano-4-hydroxycinnamic acid (CHCA) matrix. Data processing was done with MestReNova Version 8.1.

Analytical RP-HPLC was carried out using a Perkin Elmer Series 200 lc pump fitted with a series 200 UV/Vis detector and autosampler using a SB-Analytical ODH-S optimal column (100 × 1.6 mm, 3.5 μm); flow rate 1 ml min^{-1}; λ = 220 nm, linear gradient elution 0-100% of solvent B over 30 min (*A* = 0.05% TFA, 95% H_2O, 5% MeCN, *B* = 0.03% TFA, 5% H_2O, 95% MeCN).

2. Biological Assays

Antibacterial MIC determination

Escherichia coli K-12 wild-type strain (W3110 / ATCC27325, F^-, λ^-, *rpoS(Am)*, *rph-1*, *Inv*(*rrnD-rrnE*)), *Pseudomonas aeruginosa* PA01 (ATCC 15692) *Staphylococcus aureus* (3R7089 strain Oxford / ATCC9144) and *Staphylococcus epidermidis* (laboratory strain from clinical isolate) were selected for bacteriological studies as representative Gram-negative (*E. coli* and *P. aeruginosa*) and Gram-positive (*S. aureus* and *S. epidermidis*) species. Bacterial cultures were prepared by streaking bacterial strains onto LB agar plates with an inoculation loop and incubated overnight at 37 °C. A single colony was selected and placed in 5 mL of Iso-sensitest broth (Oxoid, ThermoScientific) and incubated with shaking for 16-18 h at 37 °C to provide liquid cultures for testing.

MIC values were obtained according to the protocol described by J. M. Andrews *et al.*[1] and were conducted in 96-well plates (Sarstedt). Bacteria were grown from overnight cultures in Iso-sensitest broth to an A_{650nm} of 0.07 equivalent to a 0.5 MacFarland standard (240 μM $BaCl_2$ in 0.18 M H_2SO_4). This culture was diluted ten-fold with Iso-sensitest broth before use. Peptoids were initially dissolved in DMSO (5 mM) and diluted further in Iso-sensitest broth to achieve a concentration range of 4 – 200 μM using 2-fold serial dilutions. 50 μl of inoculum and 50 μl of peptoid solution were added to each test well (final concentration range of 2 – 100 μM). Experiments were performed in triplicate. A positive control for bacterial growth contained only the inoculum and Iso-sensitest broth. Other controls contained the inoculum and serial dilutions of ampicillin (from 250 μg/mL to 2 μg/mL), serial dilutions of DMSO and the inoculum to confirm no inhibitory effect on bacterial growth, and Iso-sensitest broth alone as a sterile control. The MIC was defined as the lowest concentration which completely inhibited bacterial growth after incubation at 37 °C for 16 h with shaking. Quantitative data was attained from absorbance values using a Biotek Synergy H4 plate reader.

Cytotoxicity assay with HepG2

Cytotoxicity analyses were performed in 96-well plates (Costar, Fisher Scientific) using alamarBlue® (Invitrogen) for cell viability detection using a modified protocol as previously described. The HepG2 cells were grown at 37 °C, 5% CO_2 in DMEM high glucose supplemented with heat-inactivated foetal bovine sera (FBS, 10%; Biosera Ltd) and penicillin/streptomycin (P/S, 1%). Cells were counted using a Neubauer Improved Haemocytometer. HepG2 cells were seeded 1 day prior to treatment in 96 well plates at a concentration of $2x10^5$ cells/mL in 100 µL of medium ($2x10^4$ cells/well). Then cells were pre-incubated with the compounds in triplicate (5 mM stock solutions in DMSO diluted from 100 µM to 3 µM; untreated cells with DMSO as a negative control) in 50 µl of the media for 1 hour. Afterwards, 40 µL were removed from each well before the addition of 90 µL of the media, followed by incubation for 24 hours at 37 °C, 5% CO_2. Then, 10 µL of alamarBlue® (Invitrogen) was added to each well before a 2 hour incubation prior to assessing cell viability using a fluorescent plate reader (Biotek; Ex 560 nm / Em 600 nm). All data was measured in triplicate on a minimum of two occasions to ensure a robust data set was collected. The ED_{50} values were calculated from the dose response results achieved from the serial dilutions.

Skin Cell Toxicity assay with HaCaT

Cytotoxicity analyses were performed in 96-well plates (Costar, Fisher Scientific) using alamarBlue® (Invitrogen) for cell viability detection. HaCaT cells were subcultured at 37 °C, 5% CO_2 in DMEM high glucose supplemented with heat-inactivated foetal bovine sera (FBS, 10%; Biosera Ltd) and penicillin/streptomycin (P/S, 1%). Cells were counted using a Neubauer Improved Haemocytometer. HaCaT cells were seeded in the plates 24 hours prior to treatment in 96 well plates at a concentration of $2x10^5$ cells/mL in 100 µL of medium ($2x10^4$ cells/well). Empty wells were filled with 100 µL PBS. After 24 hours, cells were incubated with the compounds in a dilution series in triplicate from 2 – 100 µM (5 mM stock solutions in DMSO diluted from 100 µM to 3 µM; Amphotericin B was used as a positive control and untreated cells with DMSO as a negative control) in 50 µL of the media for 1 hour. Afterwards, 40 µl was removed from each well, 90 µL of medium was added to each well and the cells incubated for 24 hours at 37 °C, 5% CO_2. 10 µL of alamarBlue® (Invitrogen) was added to each well before incubation for 1 hour. Cell viability was determined using a fluorescent plate reader (Synergy H4; Ex 540 nm / Em 620 nm). All data was measured in triplicate on a minimum of two

occasions to ensure a robust data set was collected. The ED_{50} values were calculated from the dose response results achieved from the serial dilutions.

3. Biological Data

Peptoid Sequence		HPLC RT (min)	ED$_{50}$ (μM)		MIC (μM)				Selectivity Index HaCaT				Selectivity Index HepG2				Average Selectivity Index			
			HaCaT	*HepG2*	*E.coli*	*P.aeruginosa*	*S.aureus*	*S.epidermidis*	*E.coli*	*P.aeruginosa*	*S.aureus*	*S.epidermidis*	*E.coli*	*P.aeruginosa*	*S.aureus*	*S.epidermidis*	*E.coli*	*P.aeruginosa*	*S.aureus*	*S.epidermidis*
(*N*ah*N*phe*N*phe)$_4$	**1**	15.7	100	100	13	100	2	6	8	1	**50**	17	8	1	**50**	17	8	1	50	17
(*N*ah*N*phe*N*phe)$_3$	**2**	15.2	100	100	50	100	6	3	2	1	17	33	2	1	17	33	2	1	17	33
(*N*ah*N*phe*N*phe)$_2$	**3**	14.1	100	100	100	100	100	100	1	1	1	1	1	1	1	1	1	1	1	1
(*N*Lys*N*phe*N*phe)$_4$	**4**	16.0	36	100	13	50	3	2	3	1	12	18	8	2	33	**50**	6	2	23	34
(*N*Lys*N*phe*N*phe)$_3$	**5**	15.4	100	100	50	100	25	6	2	1	4	17	2	1	4	17	2	1	4	17
(*N*Lys*N*phe*N*phe)$_2$	**6**	14.2	100	100	100	100	100	100	1	1	1	1	1	1	1	1	1	1	1	1
(*N*ae*N*phe*N*phe)$_4$	**7**	16.3	100	100	13	50	2	6	8	2	**50**	17	8	2	**50**	17	8	2	50	17
(*N*ae*N*phe*N*phe)$_3$	**8**	15.7	100	100	100	100	13	100	1	1	8	1	1	1	8	1	1	1	8	1
(*N*ae*N*phe*N*phe)$_2$	**9**	14.0	100	100	100	100	100	100	1	1	1	1	1	1	1	1	1	1	1	1
(*N*ah*N*spe*N*spe)$_4$	**10**	17.7	23	41	25	50	2	2	1	0	12	12	2	1	21	21	2	1	16	17
(*N*ah*N*spe*N*spe)$_3$	**11**	16.8	100	100	25	100	3	2	4	1	33	**50**	4	1	33	**50**	4	1	33	50
(*N*ah*N*spe*N*spe)$_2$	**12**	15.2	100	100	100	100	100	25	1	1	1	4	1	1	1	4	1	1	1	4
(*N*Lys*N*spe*N*spe)$_4$	**13**	16.2	20	29	25	50	2	1	1	0	10	20	1	1	15	29	1	1	13	25
(*N*Lys*N*spe*N*spe)$_3$	**14**	15.3	100	100	13	100	2	2	8	1	**50**	**50**	8	1	**50**	**50**	8	1	50	50
(*N*Lys*N*spe*N*spe)$_2$	**15**	14.4	100	100	100	100	100	25	1	1	1	4	1	1	1	4	1	1	1	4
(*N*ae*N*spe*N*spe)$_4$	**16**	18.1	26	41	100	50	2	2	0	1	13	13	0	1	21	21	0	1	17	17
(*N*ae*N*spe*N*spe)$_3$	**17**	17.3	100	100	25	100	2	13	4	1	**50**	8	4	1	**50**	8	4	1	50	8
(*N*ae*N*spe*N*spe)$_2$	**18**	15.4	100	100	100	100	100	100	1	1	1	1	1	1	1	1	1	1	1	1
(*N*Lys*N*pmb*N*pmb)$_4$	**19**	16.5	41	100	100	100	3	2	0	0	14	21	1	1	33	**50**	1	1	24	36

(*N*Lys*N*pcb*N*pcb)$_4$	**20**	20.6	18	22	100	100	25	6	0	0	1	3	0	0	1	4	0	0	1	4
(*N*Lys*N*pcb*N*pcb)$_3$	**21**	19.6	22	23	50	25	3	2	0	1	7	11	0	1	8	12	0	1	8	12
(*N*Lys*N*pfb*N*pfb)$_4$	**22**	17.5	46	30	13	25	2	1	4	2	23	46	2	1	15	30	3	2	19	38
(*N*Lys*N*pfb*N*pfb)$_3$	**23**	16.7	100	45	13	25	3	3	8	4	33	33	3	2	15	15	6	3	24	24
(*N*Lys*N*mfb*N*mfb)$_4$	**24**	16.9	25	17	25	25	6	3	1	1	4	8	1	1	3	6	1	1	4	7
(*N*Lys*N*mfb*N*mfb)$_3$	**25**	16.4	64	43	13	25	6	2	5	3	11	32	3	2	7	22	4	3	9	27
(*N*Lys*N*pfb*N*spe)$_4$	**26**	19.7	20	26	13	13	2	2	2	2	10	10	2	2	13	13	2	2	12	12
(*N*Lys*N*pfb*N*spe)$_3$	**27**	16.6	52	36	25	25	3	2	2	2	17	26	1	1	12	18	2	2	15	22
[(*N*Lys*N*pfb*N*pfb)(*N*Lys*N*spe*N*spe)]$_2$	**28**	17.6	100	55	6	50	2	1	**17**	2	**50**	**100**	9	1	28	55	13	2	39	78
(*N*Lys*N*spe*N*spe)(*N*Lys*N*pfb*N*pfb)(*N*Lys*N*spe*N*spe)	**29**	16.5	65	43	13	25	3	2	5	3	22	33	3	2	14	22	4	3	21	28
(*N*amy*N*spe*N*spe)[(*N*Lys*N*spe*N*spe)]$_3$	**30**	19.3	12	15	50	50	2	2	0	0	6	6	0	0	8	8	0	0	7	7
(*N*amy*N*spe*N*spe)$_2$(*N*Lys*N*spe*N*spe)$_2$	**31**	22.8	20	18	100	100	2	1	0	0	10	20	0	0	9	18	0	0	10	19
(*N*Lys*N*spe*N*spe)$_2$(*N*amy*N*spe*N*spe)(*N*Lys*N*spe*N*spe)	**32**	20.0	20	22	100	100	6	2	0	0	3	10	0	0	4	11	0	0	4	11
(*N*hArg*N*phe*N*phe)$_4$	**33**	16.2	100		6	25		2	**17**	4		**50**					17	4	-	50
(*N*hArg*N*spe*N*spe)$_4$	**34**	17.8	20	12	6	13	1	1	3	2	20	20	2	1	12	12	3	2	16	16
(*N*hArg*N*spe*N*spe)$_3$	**35**	17.2			6	50	2	2									-	-	-	-
(*N*hArg*N*mfb*N*mfb)$_4$	**36**	17.2	28	21	13	13	2	2	2	2	14	14	2	2	11	11	2	2	12	12
(*N*hArg*N*mfb*N*mfb)$_3$	**37**	16.7			6	25	2	1									-	-	-	-
(*N*hArg*N*hLeu*N*spe)$_4$	**38**	18.9			13	25	1	2									-	-	-	-
(*N*hArg*N*hLeu*N*spe)$_3$	**39**	17.3				100	3	2									-	-	-	-
[(*N*amy*N*spe*N*spe)(*N*hArg*N*spe*N*spe)]$_2$	**40**	22.4	31	24	100	100	3	6	0	0	10	5	0	0	8	4	0	0	9	5
(*N*Lys*N*spe*N*spe)$_2$(*N*hArg*N*spe*N*spe)$_2$	**41**	16.5	100		17	34	17		6	3	6						6	3	6	-
(*N*hArg*N*spe*N*spe)$_2$(*N*Lys*N*spe*N*spe)$_2$	**42**	16.9	15		17	17	17		1	1	1						1	1	1	-
(*N*Lys*N*spe*N*spe)(*N*hArg*N*spe*N*spe)(*N*Lys*N*spe*N*spe)$_2$	**43**	17.8	33		17	17	17		2	2	2						2	2	2	-
[(*N*hArg*N*spe*N*spe)(*N*Lys*N*spe*N*spe)]$_2$	**44**	16.5	33		17	67	17		2	0	2						2	0	2	-

4. Characterisation

Characterisation of building blocks and peptoids used in this study

The following table shows the amine sub-monomers used to synthesize the peptoids described in this paper.

Monomer	Chemical structure	Amine sub-monomer
*N*Lys<br><br>***N*-(4-aminobutyl) glycine**	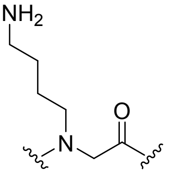	*N*-Boc-1,4-diaminobutane
*N*ah<br><br>***N*-(4-aminohexyl) glycine**	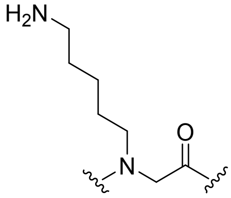	*N*-Boc-1,4-diaminohexane
*N*ae<br><br>***N*-(4-aminoethyl) glycine**	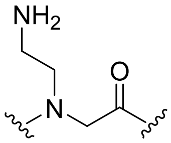	*N*-Boc-1,4-diaminoethane
*N*amy<br><br>***N*-(pentyl) glycine**	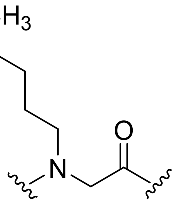	amylamine
*N*hArg<br><br>***N*-(4-guanidinopropyl) glycine**	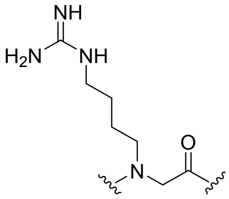	*n/a*<br><br>*post synthetic modification to unprotected 1,4-diaminobutane*
*N*nArg<br><br>***N*-(2-guanidinopropyl) glycine**	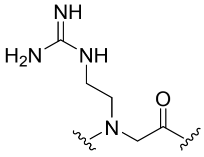	*n/a*<br><br>*post synthetic modification to unprotected 1,2-diaminoethane*
*N*phe<br><br>***N*-(phenylmethyl) glycine**	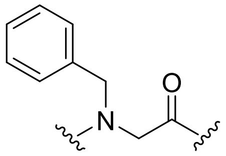	benzylamine

*N*spe<br><br>**N(*S*-phenylethyl) glycine**	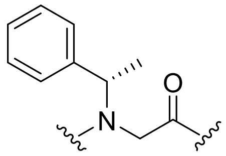	*(S)*-(−)-α-Methylbenzylamine
*N*pmb<br><br>***N*-(4-methoxyphenylmethyl) glycine**	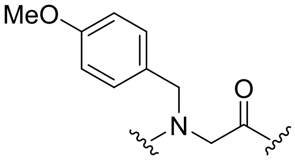	4-methoxybenzylamine
*N*pfb<br><br>***N*-(4-fluoro phenylmethyl) glycine**	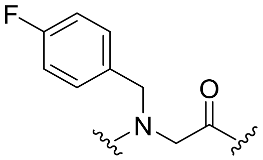	4-fluorobenzylamine
*N*mfb<br><br>***N*-(3-fluoro phenylmethyl) glycine**	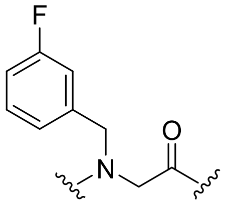	3-fluorobenzylamine
*N*pcb<br><br>***N*-(4-chloro phenylmethyl) glycine**	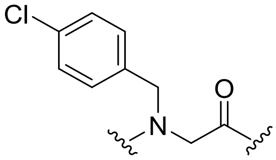	4-chlorobenzylamine
*N*hLeu<br><br>***N*-(isopentyl) glycine**	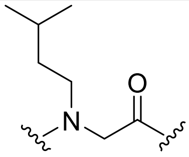	isopentylamine

Table 1. The abbreviations used for the peptoid monomers used in this study, and the amines that they are derived from.

Accurate Mass and Analytical RP-HPLC Data

Accurate mass data for the peptoids tested are shown in *Table 2.* Data were obtained for the $[M+2H]^{2+}$ ion if compounds were too large to study the $[M+H]^{+}$ ion.

Analytical HPLC retention times are also tabulated; analytical HPLC gradient: 0 – 100% solvent B over 30 min at 220 nm (where solvent A = 95% H_2O, 5% MeCN, 0.05 % TFA; solvent B = 95% MeCN, 5% H_2O, 0.03% TFA) with the column oven set to 40˚C. Retention time calculated from the middle of the peak. Most compounds were obtained with purity >95%, chromatograms can be found after *Table 2*.

	Sequence	Mass Calculated	Mass Observed	Chemical Formula	HPLC R_T ***(min)***
1	(*N*ah*N*phe*N*phe)$_4$	910.0473	910.0468	$C_{104}H_{139}N_{17}O_{12}$	15.7
2	(*N*ah*N*phe*N*phe)$_3$	684.9157	684.9141	$C_{78}H_{105}N_{13}O_9$	15.2
3	(*N*ah*N*phe*N*phe)$_2$	918.5605	918.5634	$C_{52}H_{71}N_9O_6$	14.1
4	(*N*Lys*N*phe*N*phe)$_4$	853.9847	853.9835	$C_{96}H_{123}N_{17}O_{12}$	16.0
5	(*N*Lys*N*phe*N*phe)$_3$	642.8688	642.8666	$C_{72}H_{93}N_{13}O_9$	15.4
6	(*N*Lys*N*phe*N*phe)$_2$	431.7529	431.7513	$C_{48}H_{63}N_9O_6$	14.2
7	(*N*ae*N*phe*N*phe)$_4$	797.9221	797.9189	$C_{88}H_{107}N_{17}O_{12}$	16.3
8	(*N*ae*N*phe*N*phe)$_3$	600.8218	600.8185	$C_{66}H_{81}N_{13}O_9$	15.7
9	(*N*ae*N*phe*N*phe)$_2$	806.4354	806.4370	$C_{44}H_{55}N_9O_6$	14.0
10	(*N*ah*N*spe*N*spe)$_4$	966.6115	966.6127	$C_{112}H_{155}N_{17}O_{12}$	17.7
11	(*N*ah*N*spe*N*spe)$_3$	726.9627	726.9601	$C_{84}H_{117}N_{13}O_9$	16.8
12	(*N*ah*N*spe*N*spe)$_2$	974.6232	974.6246	$C_{56}H_{79}N_9O_6$	15.2
13	(*N*Lys*N*spe*N*spe)$_4$	910.0473	910.0483	$C_{104}H_{139}N_{17}O_{12}$	16.2
14	(*N*Lys*N*spe*N*spe)$_3$	684.9157	684.9142	$C_{78}H_{105}N_{13}O_9$	15.3
15	(*N*Lys*N*spe*N*spe)$_2$	459.7842	459.7801	$C_{52}H_{71}N_9O_6$	14.4
16	(*N*ae*N*spe*N*spe)$_4$	853.9847	853.9847	$C_{96}H_{123}N_{17}O_{12}$	16.0
17	(*N*ae*N*spe*N*spe)$_3$	642.8688	642.8660	$C_{72}H_{93}N_{13}O_9$	17.0
18	(*N*ae*N*spe*N*spe)$_2$	862.4980	862.4994	$C_{48}H_{63}N_9O_6$	14.1
19	(*N*Lys*N*pmb*N*pmb)$_4$	974.0269	974.0264	$C_{104}H_{139}N_{17}O_{20}$	16.5
20	(*N*Lys*N*pmb*N*pmb)$_3$	1464.7931	1464.7937	$C_{78}H_{105}N_{13}O_{15}$	15.6
21	(*N*Lys*N*pmb*N*pmb)$_2$	982.5402	982.5395	$C_{52}H_{71}N_9O_{10}$	14.5
22	(*N*Lys*N*pcb*N*pcb)$_4$	989.8288	989.8279	$C_{96}H_{115}Cl_8N_{17}O_{12}$	20.6
23	(*N*Lys*N*pcb*N*pcb)$_3$	1488.4960	1488.4960	$C_{72}H_{87}Cl_6N_{13}O_9$	19.6
24	(*N*Lys*N*pcb*N*pcb)$_2$	998.3420	998.3422	$C_{48}H_{59}Cl_4N_9O_6$	17.9

25	(*N*Lys*N*pfb*N*pfb)$_4$	1850.8861	1850.8865	$C_{96}H_{115}F_8N_{17}O_{12}$	17.5
26	(*N*Lys*N*pfb*N*pfb)$_3$	1392.6732	1392.6732	$C_{72}H_{87}F_6N_{13}O_9$	16.7
27	(*N*Lys*N*pfb*N*pfb)$_2$	934.4603	934.4601	$C_{48}H_{59}F_4N_9O_6$	15.0
28	(*N*Lys*N*mfb*N*mfb)$_4$	925.9470	925.9431	$C_{96}H_{115}F_8N_{17}O_{12}$	16.9
29	(*N*Lys*N*mfb*N*mfb)$_3$	1392.6732	1392.6746	$C_{72}H_{87}F_6N_{13}O_9$	16.4
30	(*N*Lys*N*mfb*N*mfb)$_2$	934.4603	934.4610	$C_{48}H_{59}F_4N_9O_6$	15.1
31	(*N*Lys*N*pfb*N*spe)$_4$	917.9971	917.9981	$C_{100}H_{127}F_4N_{17}O_{12}$	19.7
32	(*N*Lys*N*pfb*N*spe)$_3$	1380.7484	1380.7505	$C_{75}H_{96}F_3N_{13}O_9$	16.6
33	(*N*Lys*N*pfb*N*spe)$_2$	926.5104	926.5111	$C_{50}H_{65}F_2N_9O_6$	15.2
34	[(*N*Lys*N*pfb*N*pfb)(*N*Lys*N*spe*N*spe)]$_2$	917.9971	917.9983	$C_{100}H_{127}F_4N_{17}O_{12}$	17.6
35	(*N*Lys*N*spe*N*spe)(*N*Lys*N*pfb*N*pfb)(*N*Lys*N*spe*N*spe)	1376.7736	1376.7734	$C_{76}H_{99}F_2N_{13}O_9$	16.5
36	(*N*Lys*N*hLeu*N*spe)$_4$	842.0786	842.0757	$C_{92}H_{147}N_{17}O_{12}$	17.6
37	(*N*Lys*N*hLeu*N*spe)$_3$	1266.8706	1266.8696	$C_{69}H_{111}N_{13}O_9$	17.1
38	(*N*amy*N*spe*N*spe)(*N*Lys*N*spe*N*spe)$_3$	909.5497	909.5507	$C_{105}H_{140}N_{16}O_{12}$	19.3
39	(*N*amy*N*spe*N*spe)$_2$(*N*Lys*N*spe*N*spe)$_2$	909.0521	909.0528	$C_{106}H_{141}N_{15}O_{12}$	22.8
40	[(*N*amy*N*spe*N*spe)(*N*Lys*N*spe*N*spe)]$_2$	909.5536	909.5457	$C_{106}H_{141}N_{15}O_{12}$	22.9
41	(*N*Lys*N*spe*N*spe)$_2$(*N*amy*N*spe*N*spe)(*N*Lys*N*spe*N*spe)	909.5497	909.5483	$C_{105}H_{140}N_{16}O_{12}$	20.0
42	Cyclic (*N*Lys*N*phe*N*phe)$_2$	863.4820	863.4823	$C_{48}H_{60}N_8O_6$	13.9
43	Cyclic (*N*Lys*N*pfb*N*pfb)$_2$	917.4337	917.4358	$C_{48}H_{56}N_8O_6$	15.5
44	Cyclic (*N*Lys*N*phe)$_3$	826.4980	826.4959	$C_{45}H_{63}N_9O_6$	13.3
45	(*N*hArg*N*phe*N*phe)$_4$	938.0283	938.0276	$C_{100}H_{131}N_{25}O_{12}$	16.7
46	(*N*hArg*N*spe*N*spe)$_4$	994.0909	994.0880	$C_{108}H_{147}N_{25}O_{12}$	17.9
47	(*N*hArg*N*spe*N*spe)$_3$	1494.8890	1484.8894	$C_{81}H_{111}N_{19}O_9$	17.3
48	(*N*hArg*N*mfb*N*mfb)$_4$	1009.9906	1009.9874	$C_{10}H_{123}F_8N_{25}O_{12}$	17.3
49	(*N*hArg*N*mfb*N*mfb)$_3$	1518.7386	1518.7372	$C_{75}H_{93}F_6N_{19}O_9$	16.7
50	(*N*hArg*N*hLeu*N*spe)$_4$	926.1222	926.1262	$C_{96}H_{155}N_{25}O_{12}$	18.8
51	(*N*hArg*N*hLeu*N*spe)$_3$	1392.9360	1392.9371	$C_{72}H_{117}N_{19}O_9$	17.2
52	[(*N*amy*N*spe*N*spe)(*N*hArg*N*spe*N*spe)]$_2$	951.0739	951.0692	$C_{108}H_{145}N_{19}O_{12}$	22.4
53	(*N*Lys*N*spe*N*spe)$_2$(*N*hArg*N*spe*N*spe)$_2$	952.0691	952.0682	$C_{106}H_{143}N_{21}O_{12}$	16.6
54	(*N*hArg*N*spe*N*spe)$_2$(*N*Lys*N*spe*N*spe)$_2$	952.0691	952.0693	$C_{106}H_{143}N_{21}O_{12}$	16.9
55	(*N*Lys*N*spe*N*spe)(*N*hArg*N*spe*N*spe)(*N*Lys*N*spe*N*spe)$_2$	931.0582	931.0579	$C_{105}H_{141}N_{19}O_{12}$	17.8
56	[(*N*hArg*N*spe*N*spe)(*N*Lys*N*spe*N*spe)]$_2$	952.0691	952.0730	$C_{106}H_{143}N_{21}O_{12}$	16.6

Table 2. Accurate mass spectrometry data and retention times from analytical RP-HPLC for the peptoid library.

Analytical HPLC Traces

Analytical HPLC traces are shown below for the new compounds in the library; analytical HPLC gradient: 0 – 100% solvent B over 30 min at 220 nm (where solvent A = 95% H_2O, 5% MeCN, 0.05 % TFA; solvent B = 95% MeCN, 5% H_2O, 0.03% TFA) with the column oven set to 40˚C.

Peptoid 1 (*N*ah*N*phe*N*phe)$_4$

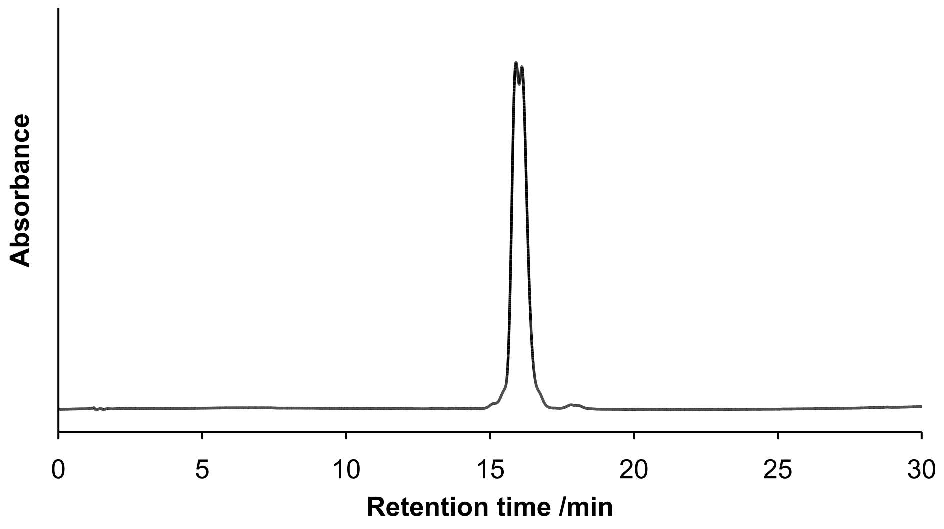

Peptoid 2 (*N*ah*N*phe*N*phe)$_3$

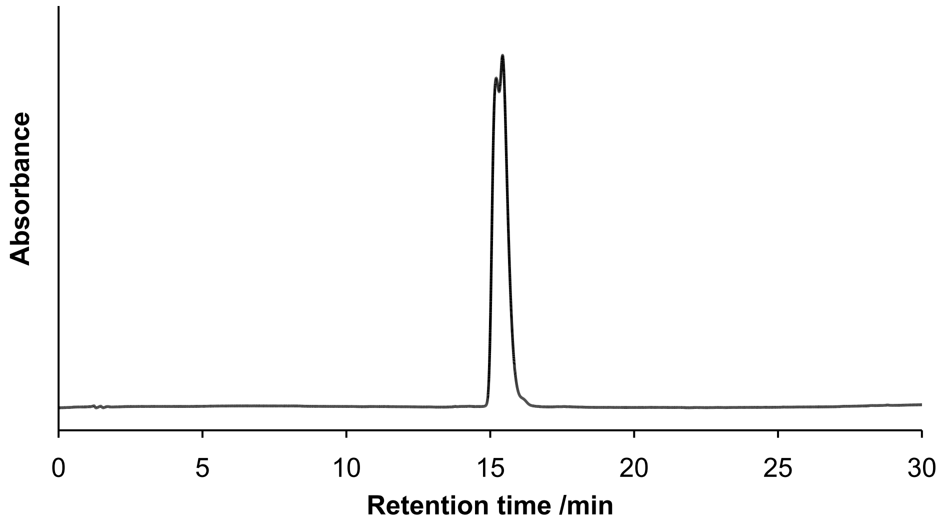

Peptoid 3 (*N*ah*N*phe*N*phe)$_2$

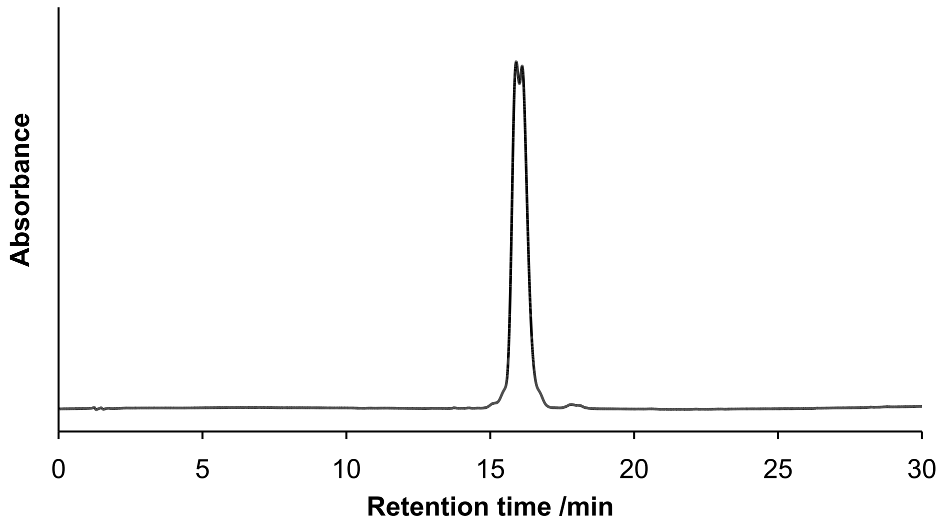

Peptoid 4 (*N*Lys*N*phe*N*phe)$_4$

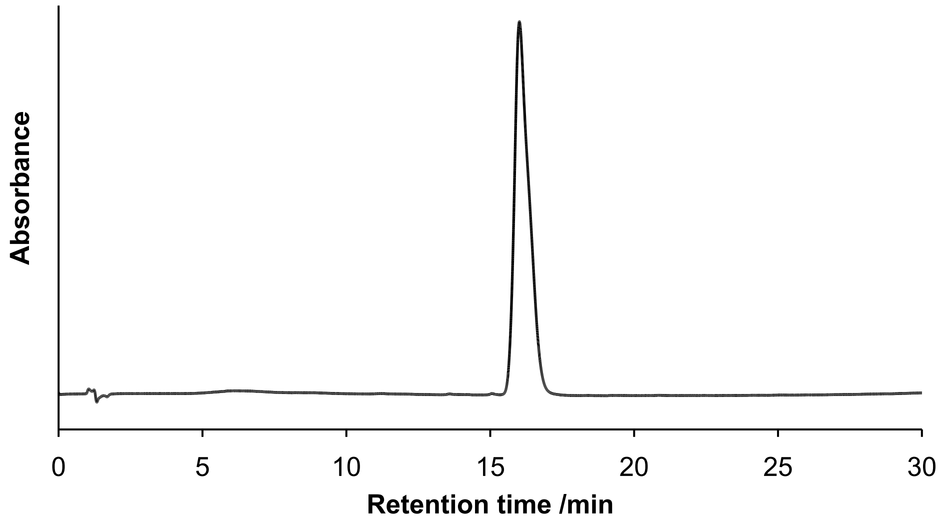

Peptoid 5 (*N*Lys*N*phe*N*phe)$_3$

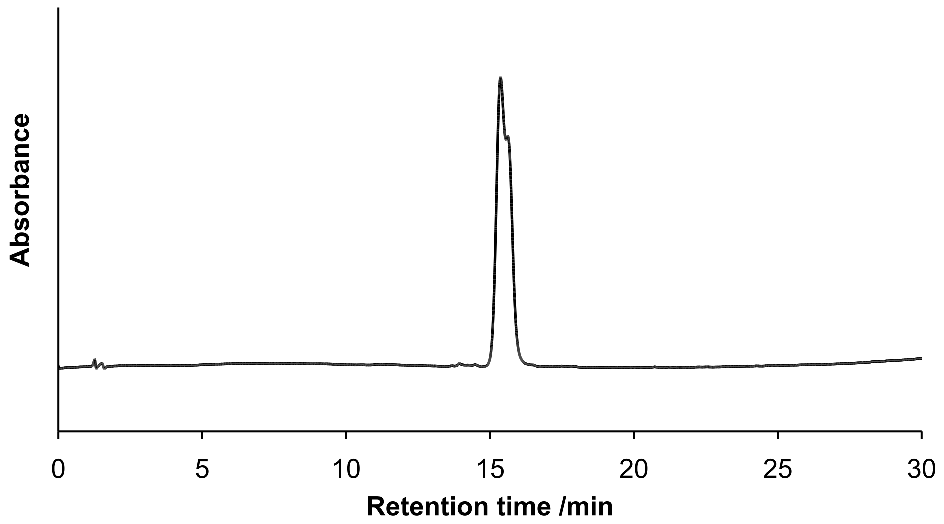

Peptoid 6 (*N*Lys*N*phe*N*phe)$_2$

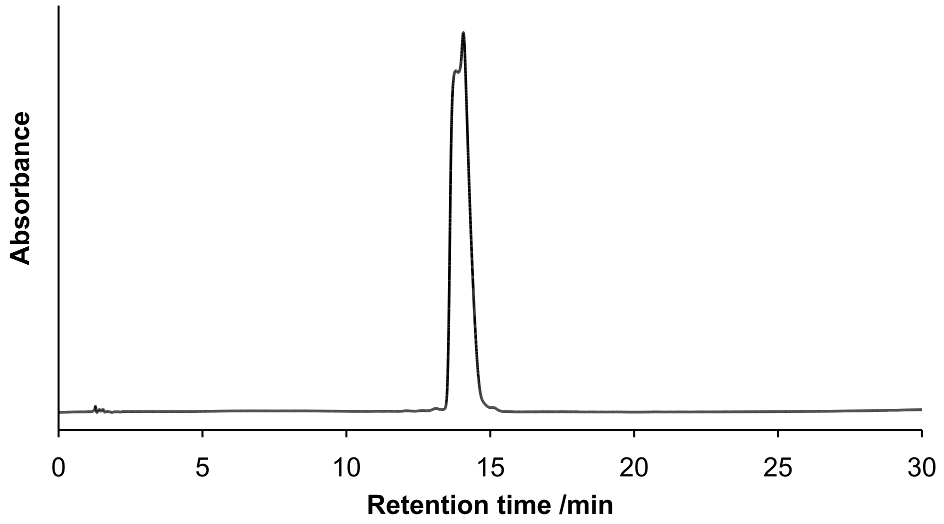

Peptoid 7 (*N*ae*N*phe*N*phe)$_4$

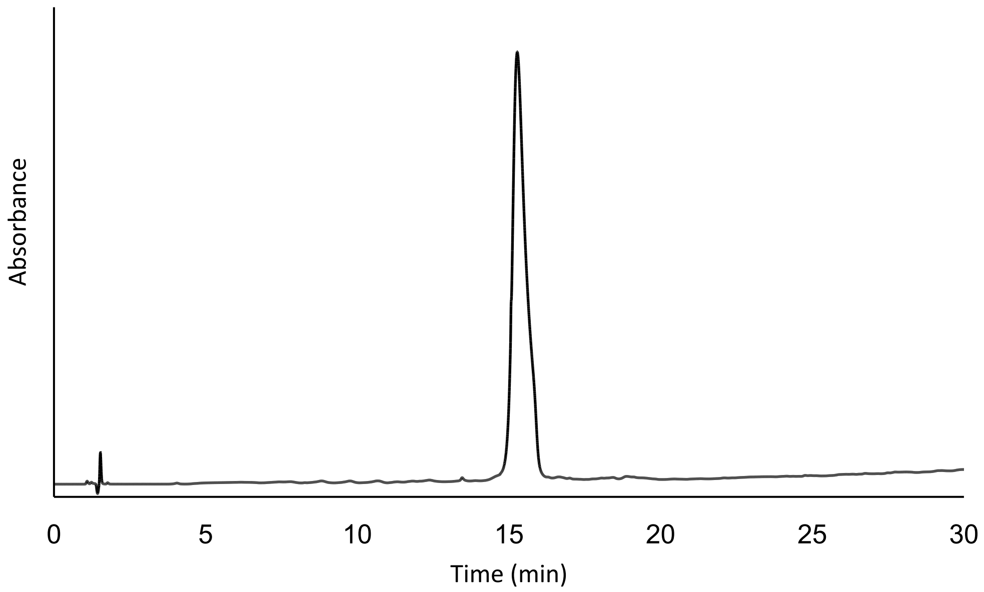

Peptoid 8 (*N*ae*N*phe*N*phe)$_3$

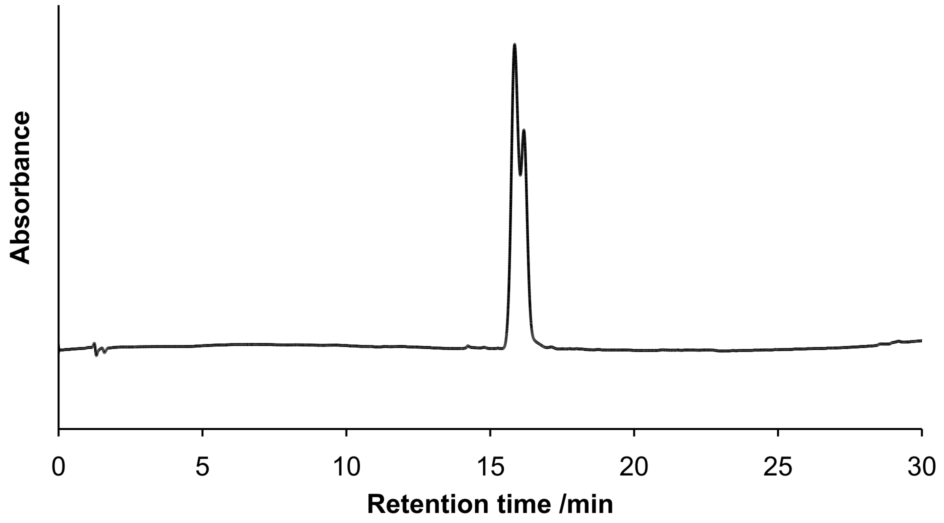

Peptoid 9 (*N*ae*N*phe*N*phe)$_2$

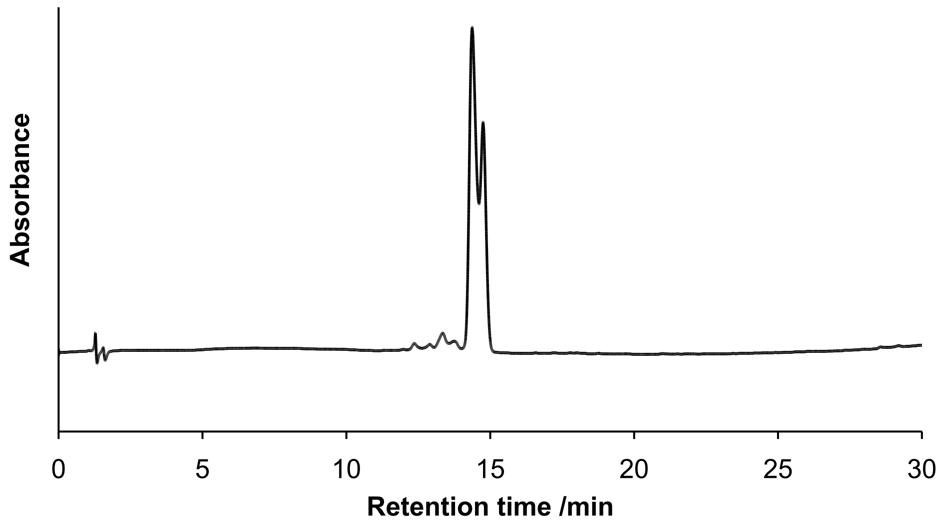

Peptoid 10 (*N*ah*N*spe*N*spe)$_4$

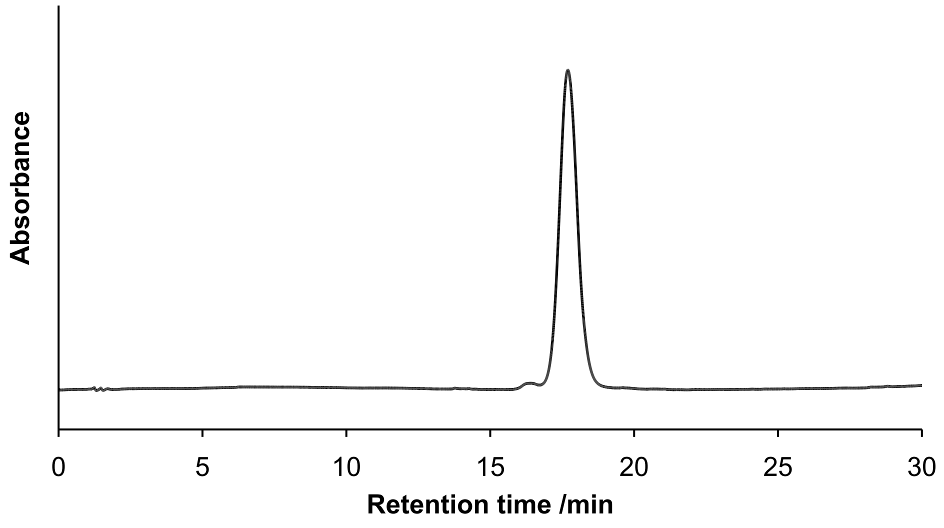

Peptoid 11 (*N*ah*N*spe*N*spe)$_3$

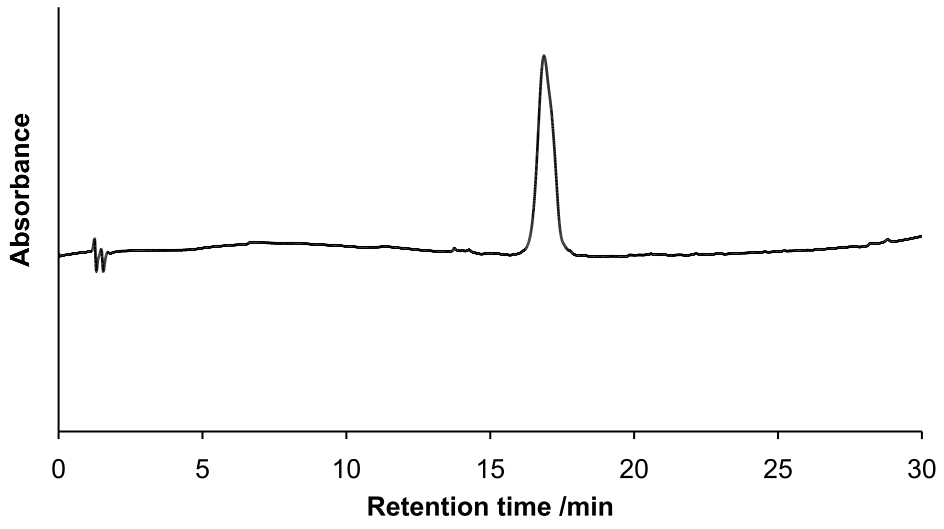

Peptoid 12 (*N*ah*N*spe*N*spe)$_2$

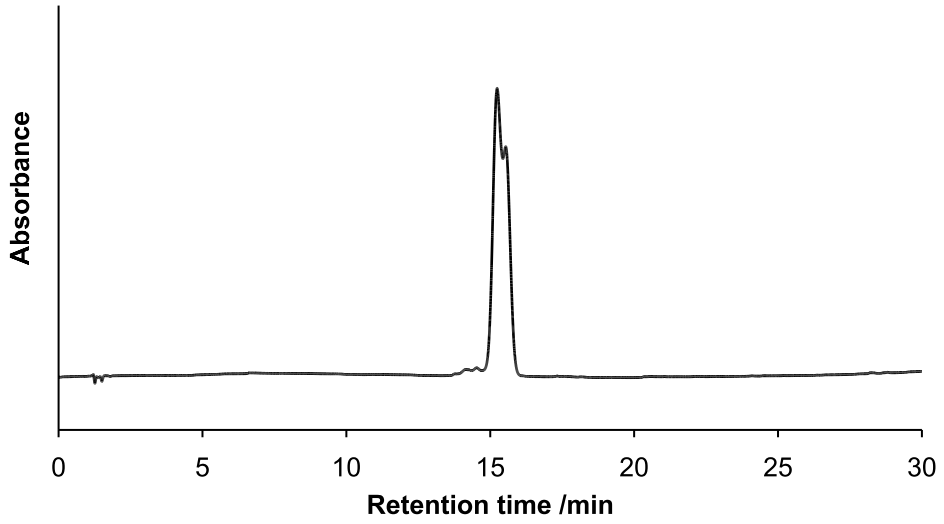

Peptoid 13 (NLys$N$spe$N$spe)$_4$

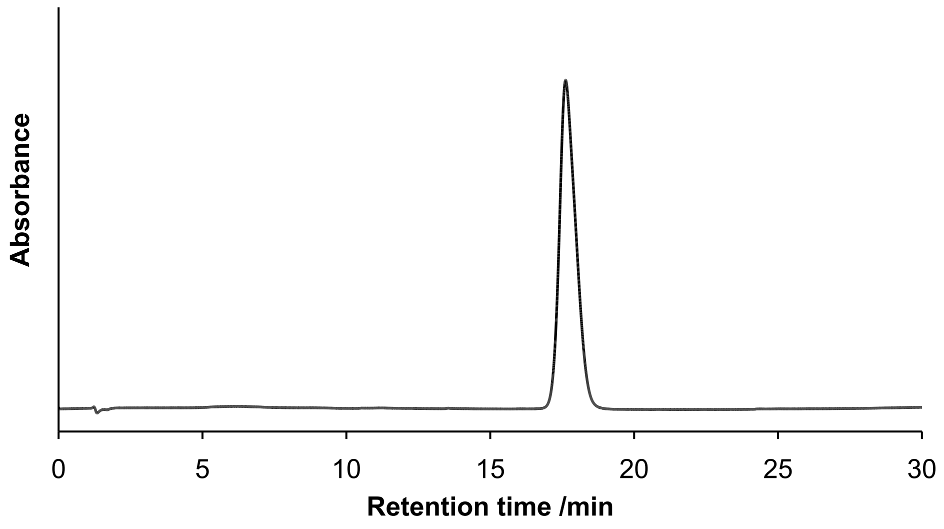

Peptoid 14 (NLys$N$spe$N$spe)$_3$

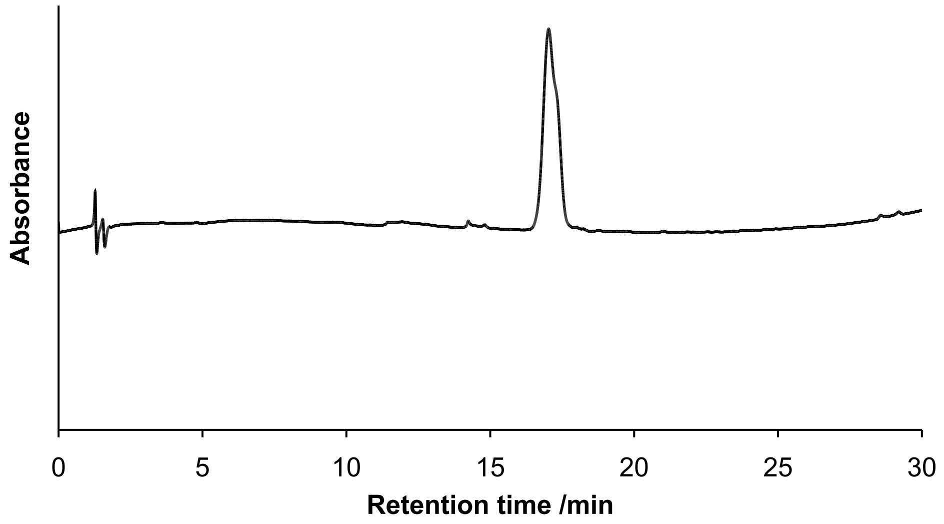

Peptoid 15 (*N*Lys*N*spe*N*spe)$_2$

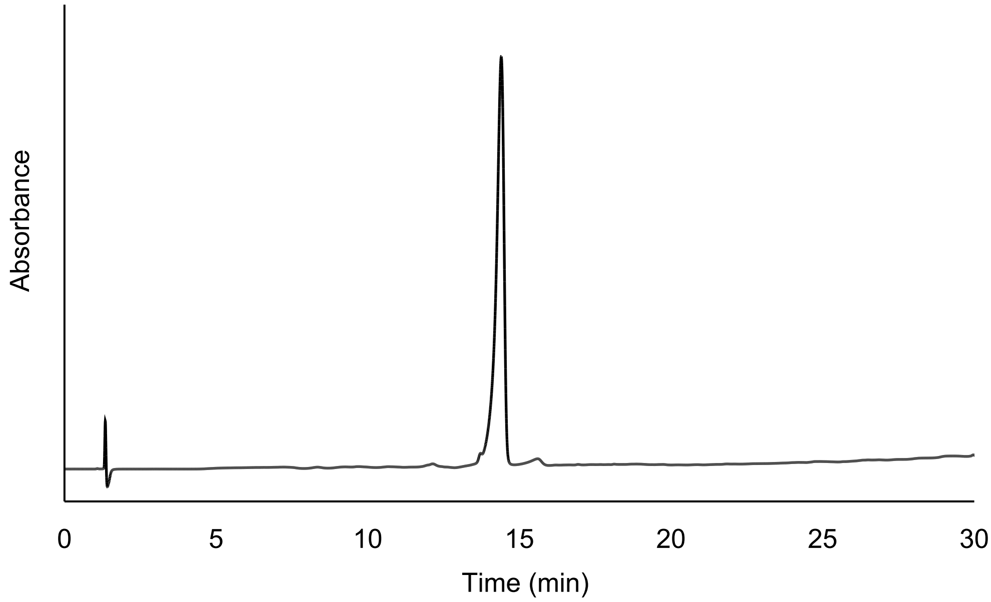

Peptoid 16 (*N*ae*N*spe*N*spe)$_4$

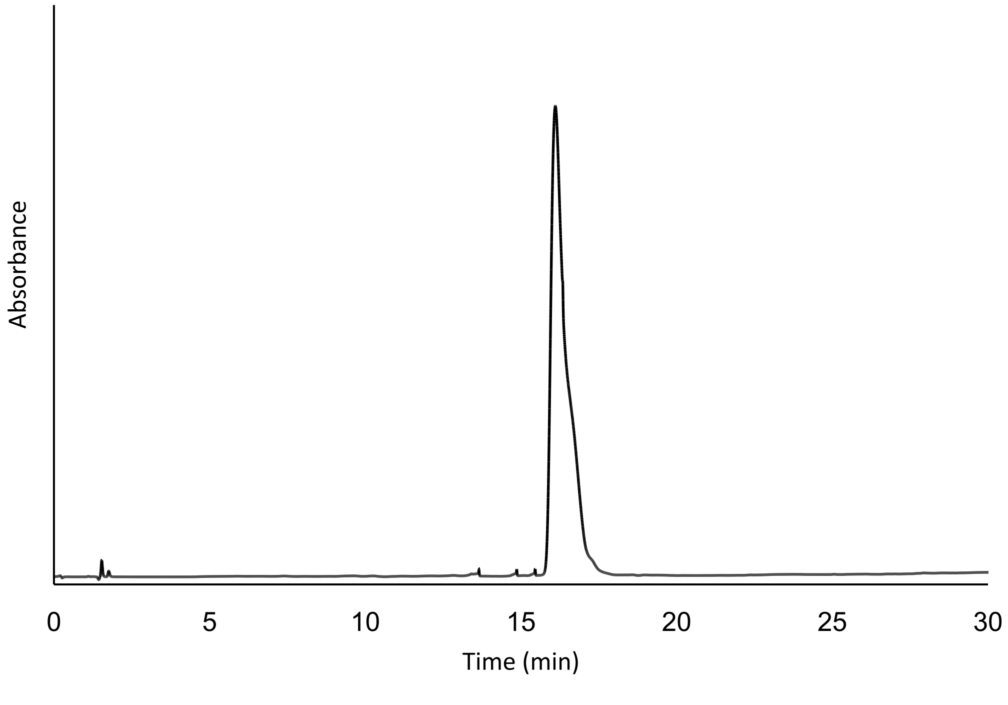

Peptoid 17 (*NaeN*spe*N*spe)$_3$**

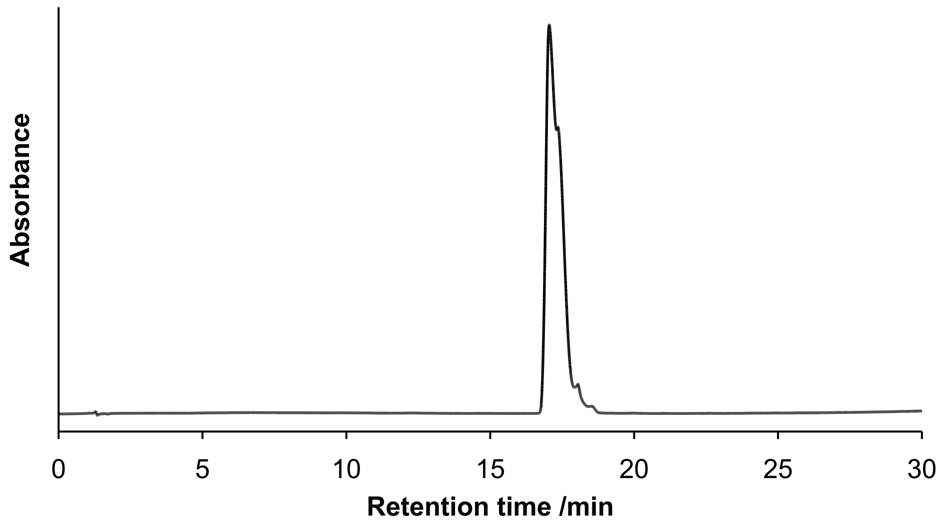

Peptoid 18 (*NaeN*spe*N*spe)$_2$**

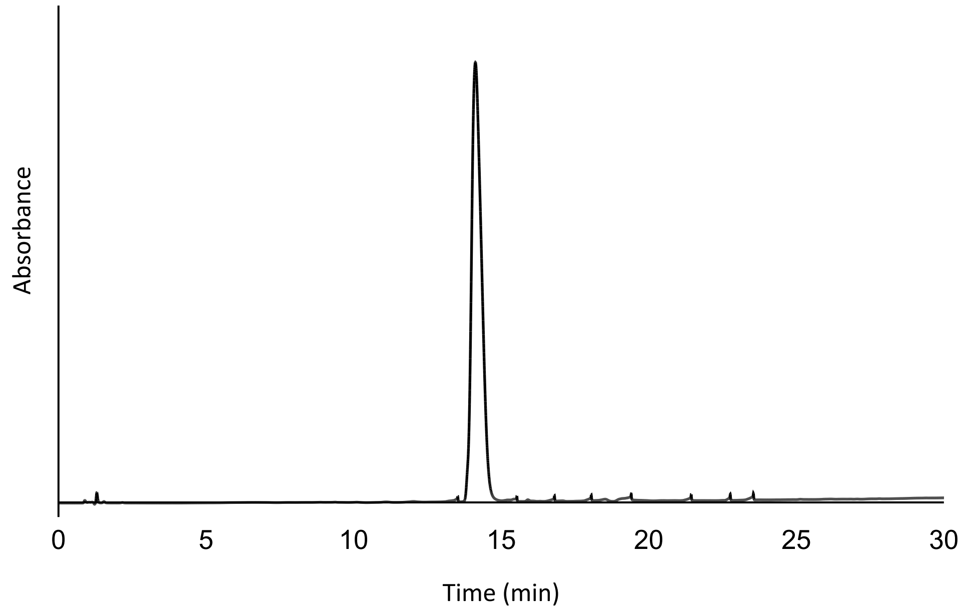

Peptoid 19 (*N*Lys*N*pmb*N*pmb)$_4$

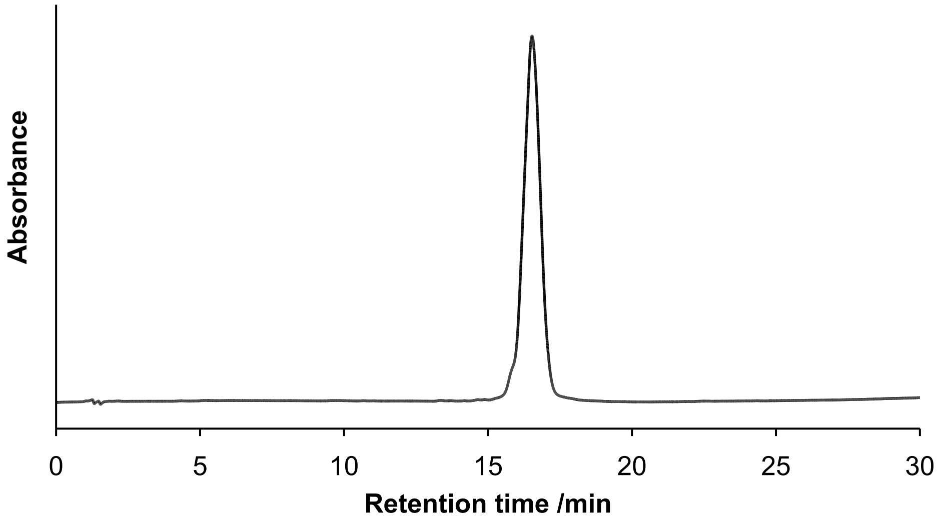

Peptoid 20 (*N*Lys*N*pcb*N*pcb)$_4$

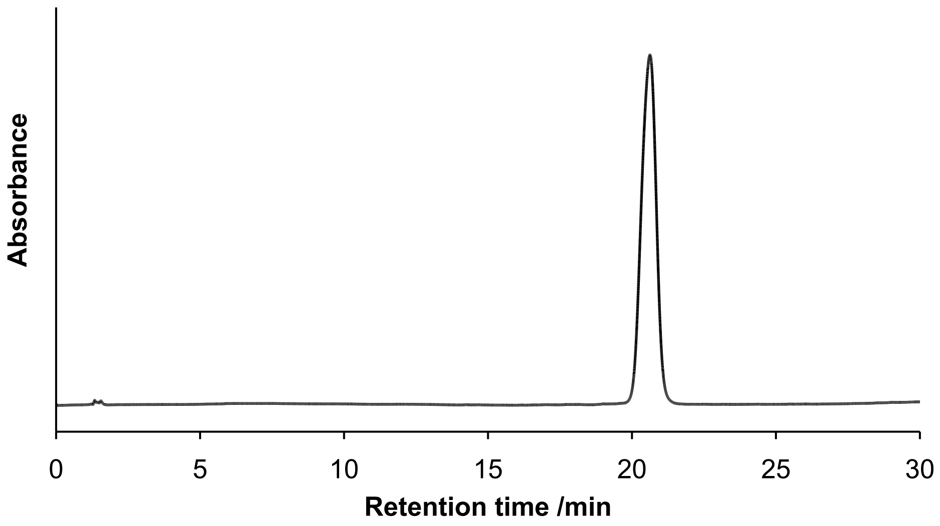

Peptoid 21 (*N*Lys*N*pcb*N*pcb)$_3$

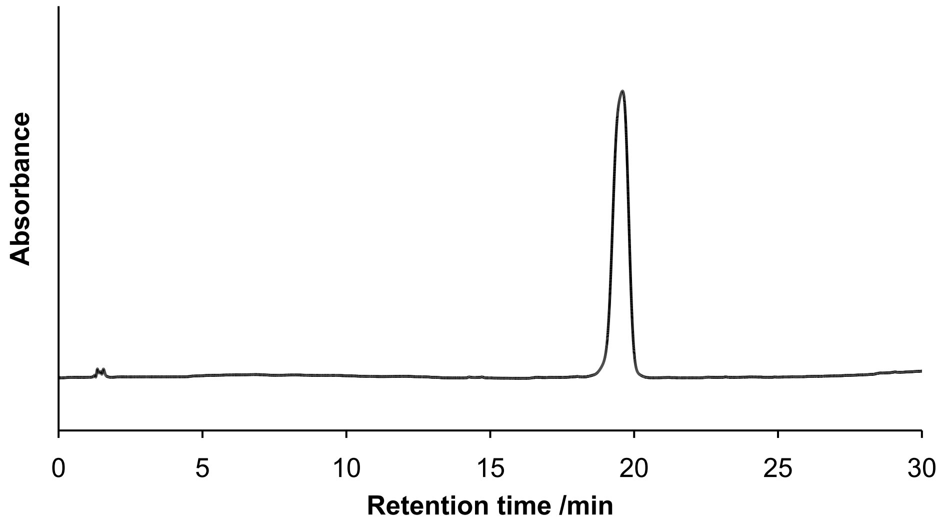

Peptoid 22 (*N*Lys*N*pfb*N*pfb)$_4$

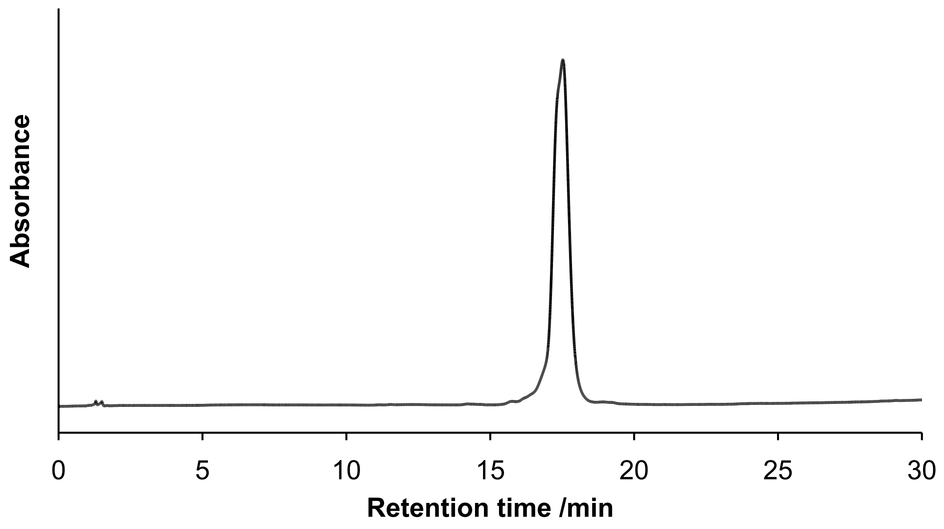

Peptoid 23 (*N*Lys*N*pfb*N*pfb)$_3$

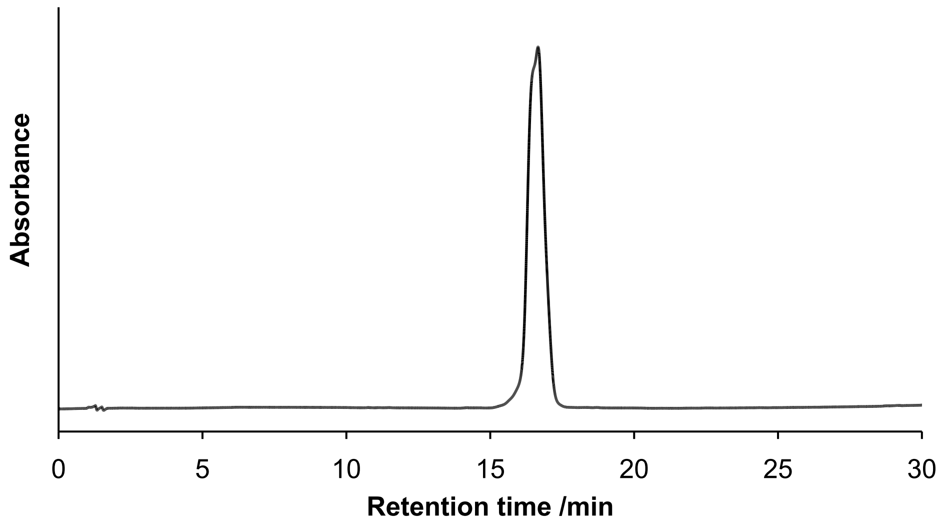

Peptoid 24 (*N*Lys*N*mfb*N*mfb)$_4$

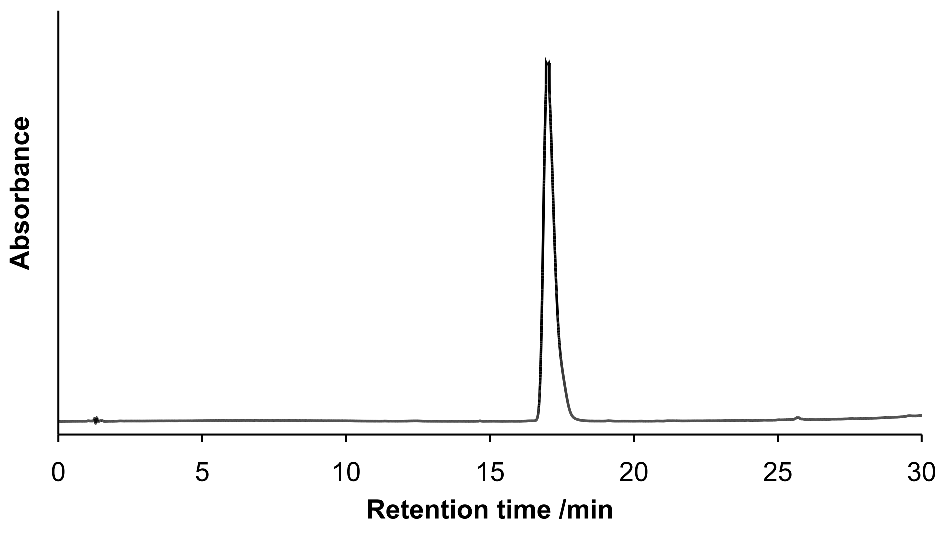

Peptoid 25 (*N*Lys*N*mfb*N*mfb)$_3$

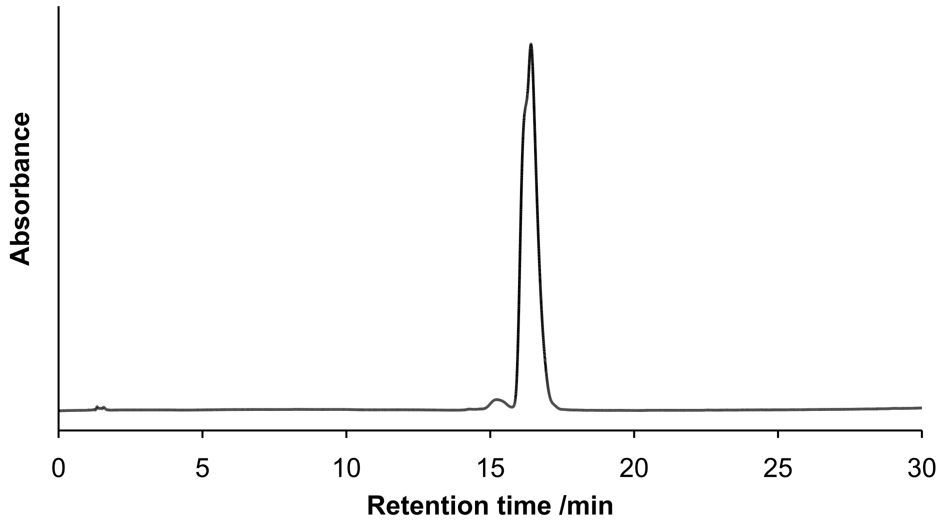

Peptoid 26 (*N*Lys*N*pfb*N*spe)$_4$

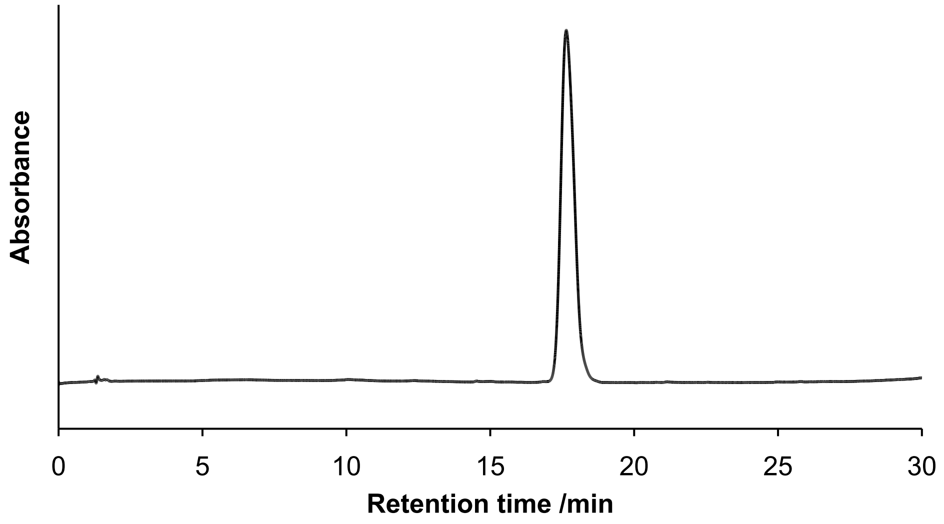

Peptoid 27 (*N*Lys*N*pfb*N*spe)$_3$

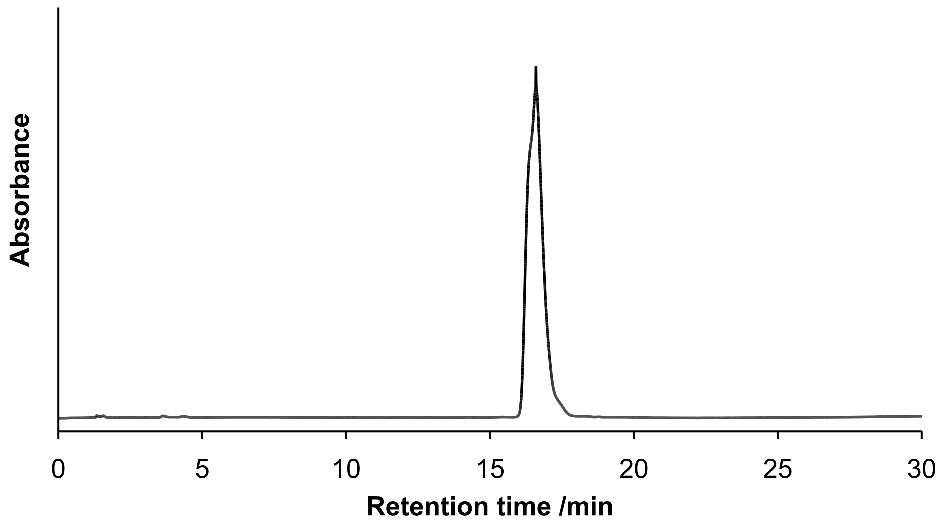

Peptoid 28 [(*N*Lys*N*pfb*N*pfb)(*N*Lys*N*spe*N*spe)]$_2$

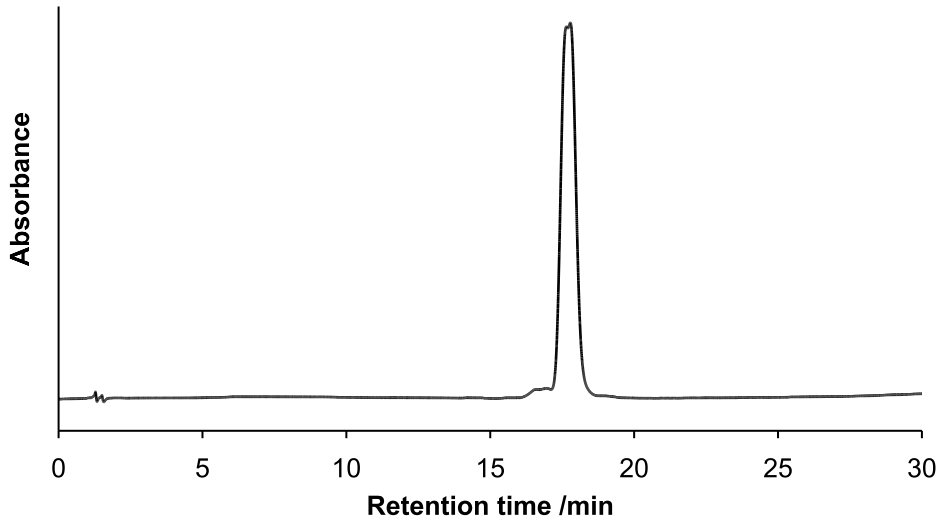

Peptoid 29 (*N*Lys*N*pfb*N*pfb)(*N*Lys*N*spe*N*spe)(*N*Lys*N*pfb*N*pfb)

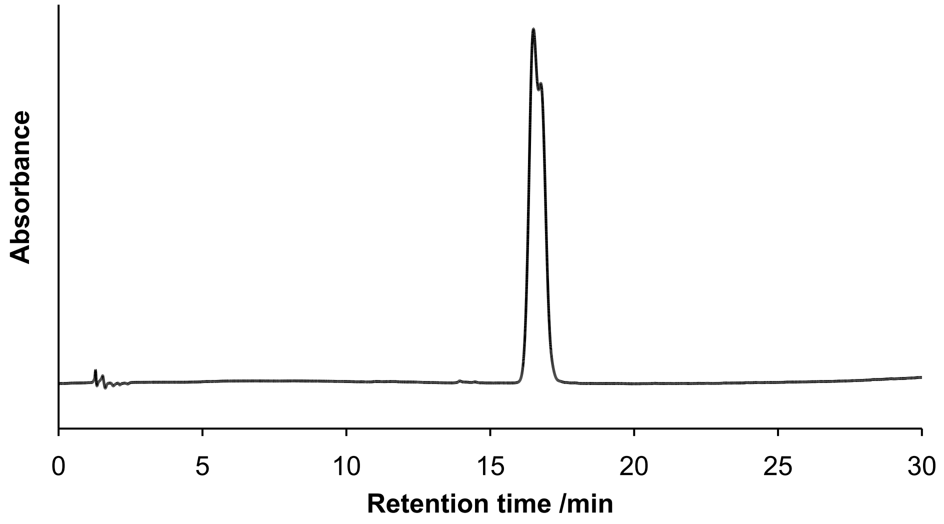

Peptoid 30 (*N*amy*N*spe*N*spe)(*N*Lys*N*spe*N*spe)$_3$

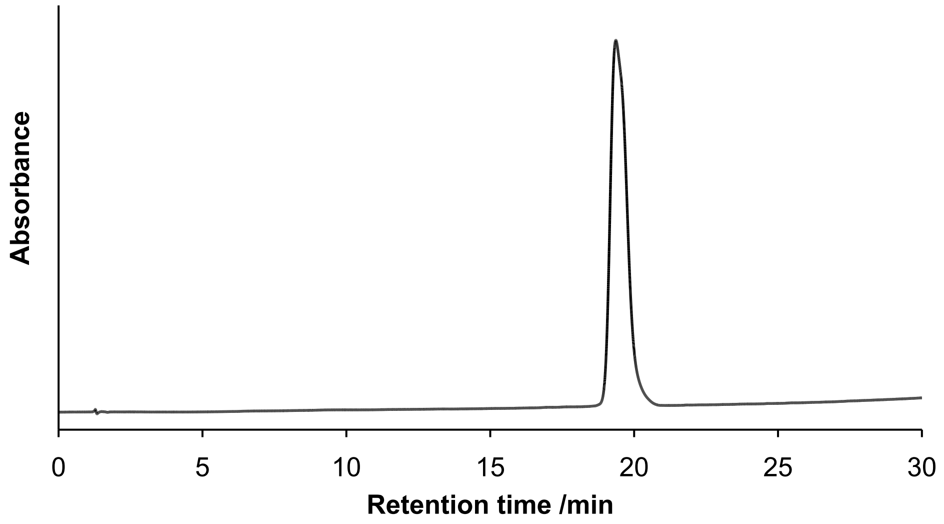

Peptoid 31 (*N*amy*N*spe*N*spe)$_2$(*N*Lys*N*spe*N*spe)$_2$

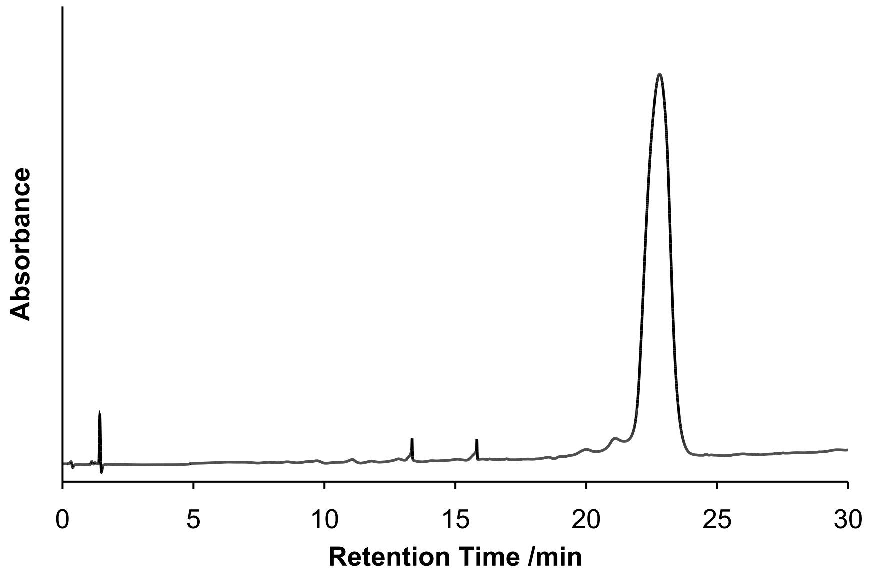

Peptoid 32 (*N*Lys*N*spe*N*spe)$_2$(*N*amy*N*spe*N*spe)(*N*Lys*N*spe*N*spe)

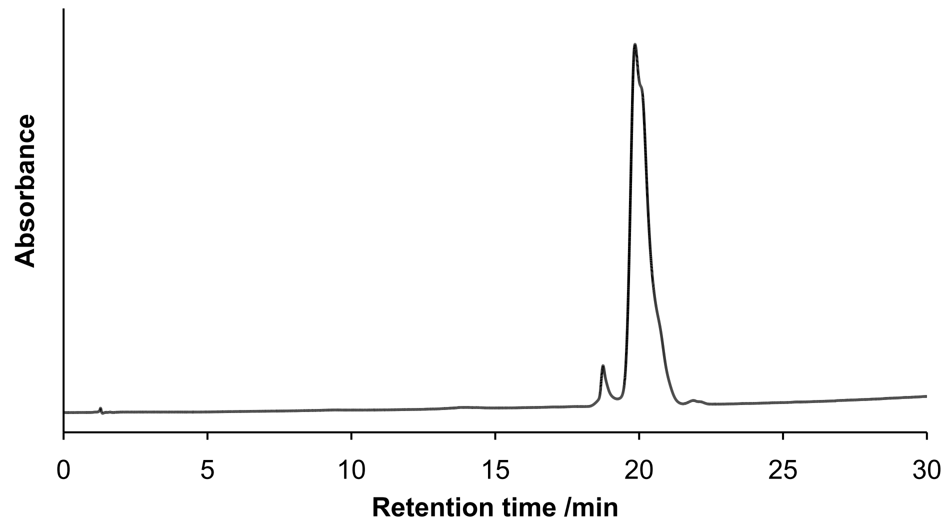

Peptoid 33 (*N*hArg*N*phe*N*phe)$_4$

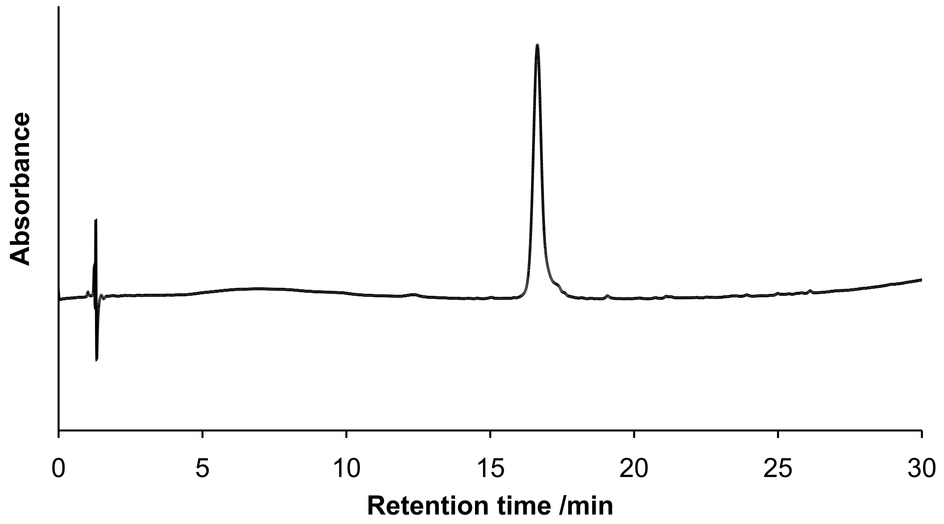

Peptoid 34 (*N*hArg*N*spe*N*spe)$_4$

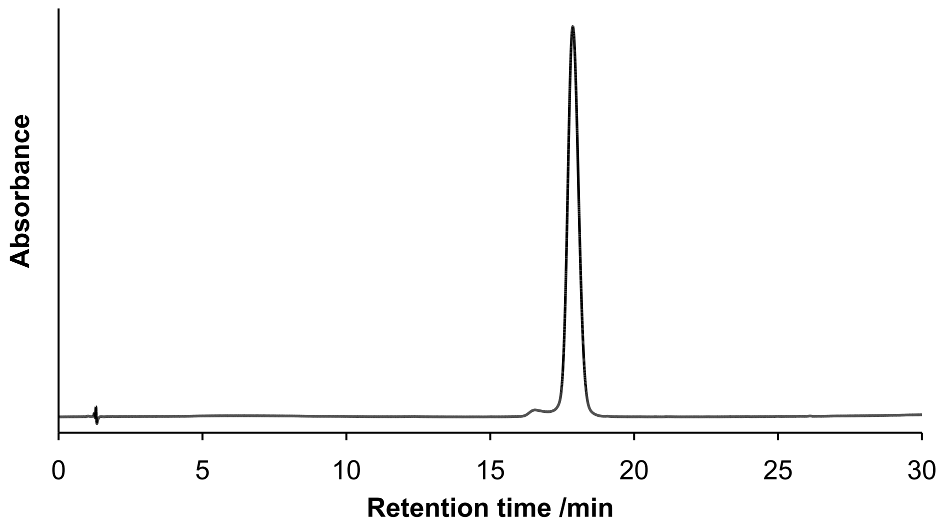

Peptoid 35 (*N*hArg*N*spe*N*spe)$_3$

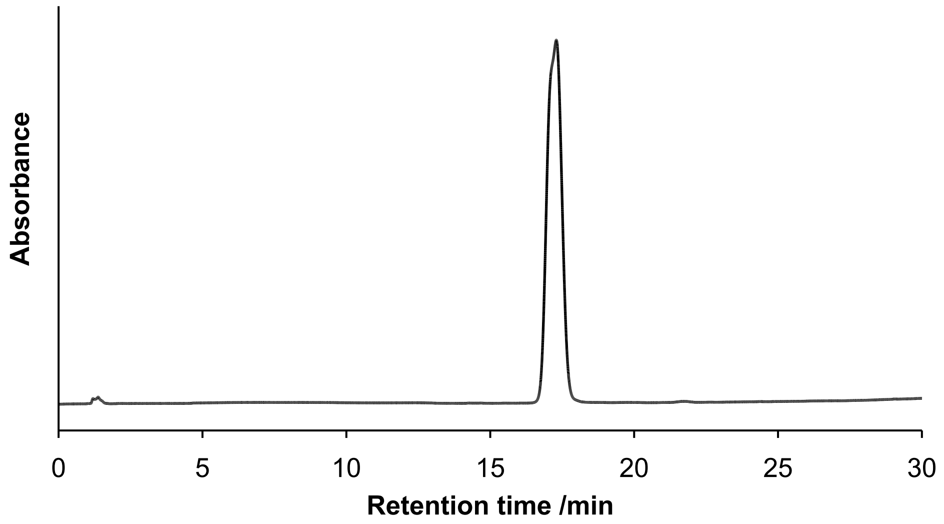

Peptoid 36 (*N*hArg*N*mfb*N*mfb)$_4$

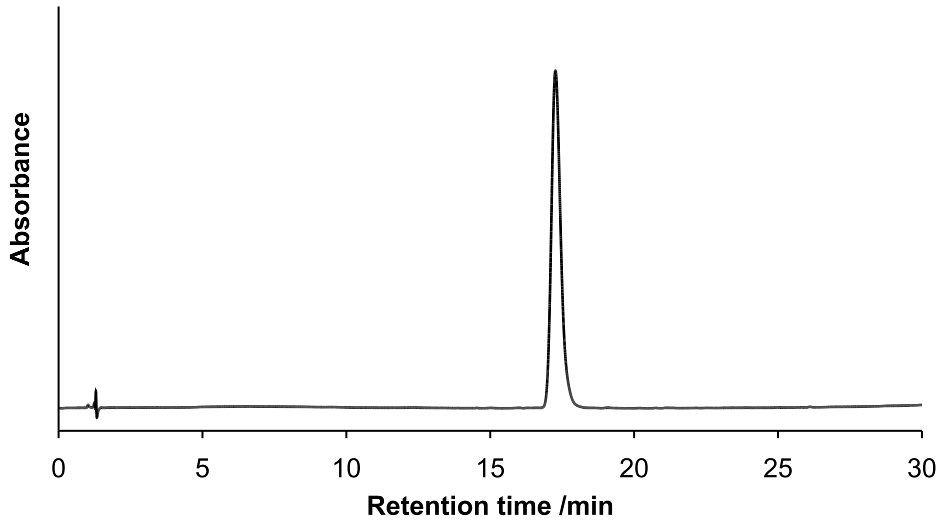

Peptoid 37 (*N*hArg*N*mfb*N*mfb)$_3$

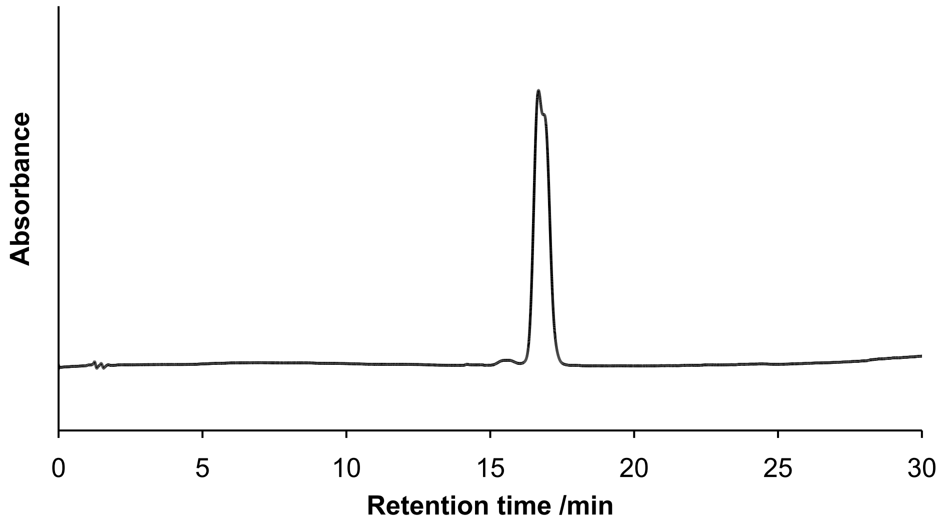

Peptoid 38 (*N*hArg*N*hLeu*N*spe)$_4$

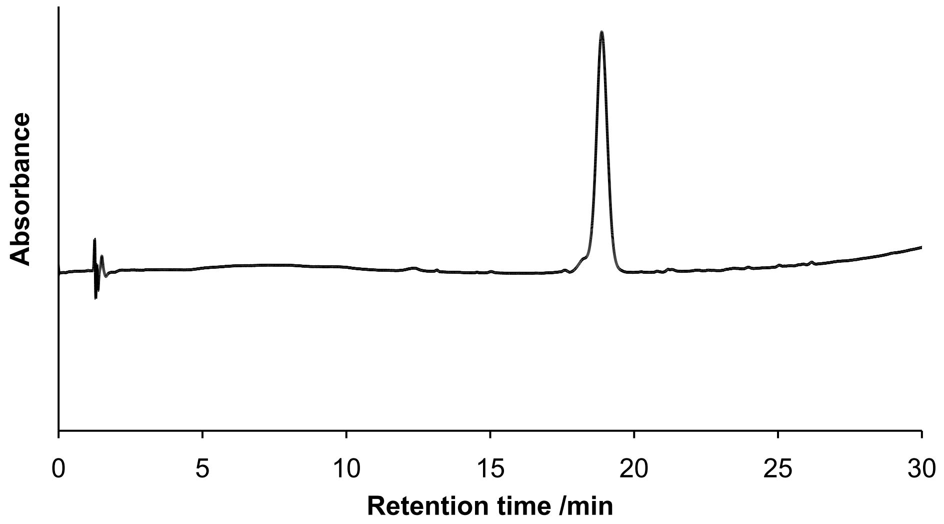

Peptoid 39 (*N*hArg*N*hLeu*N*spe)$_3$

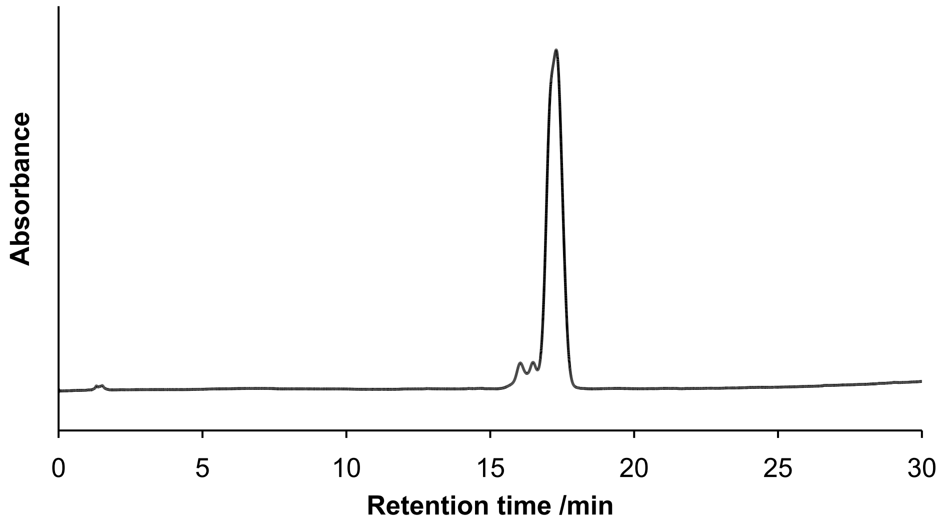

Peptoid 40 [(*N*amy*N*spe*N*spe)(*N*hArg*N*spe*N*spe)]$_2$

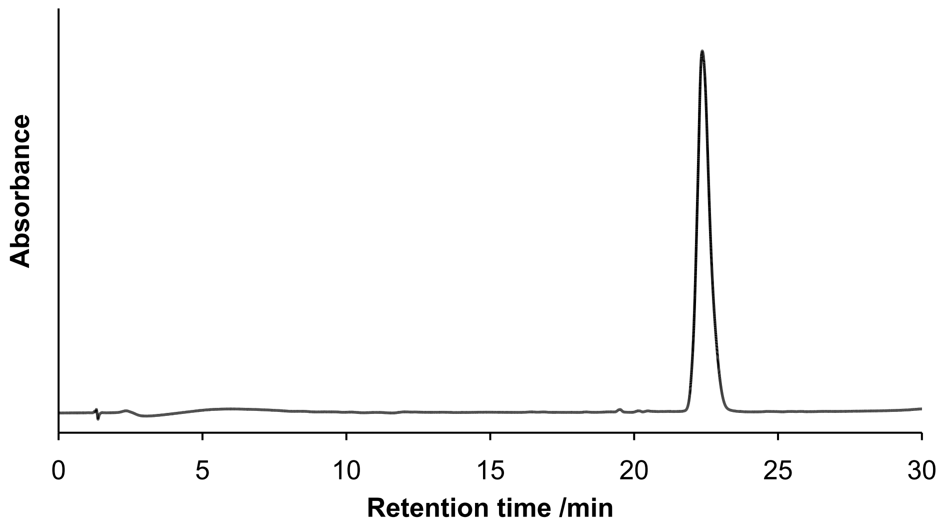

Peptoid 41 (*N*Lys*N*spe*N*spe)$_2$(*N*hArg*N*spe*N*spe)$_2$

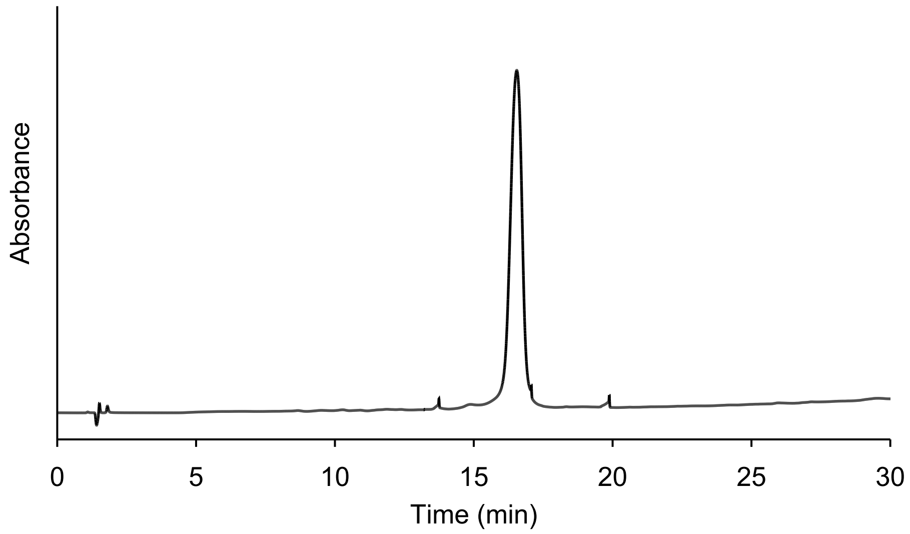

Peptoid 42 (*N*hArg*N*spe*N*spe)$_2$(*N*Lys*N*spe*N*spe)$_2$

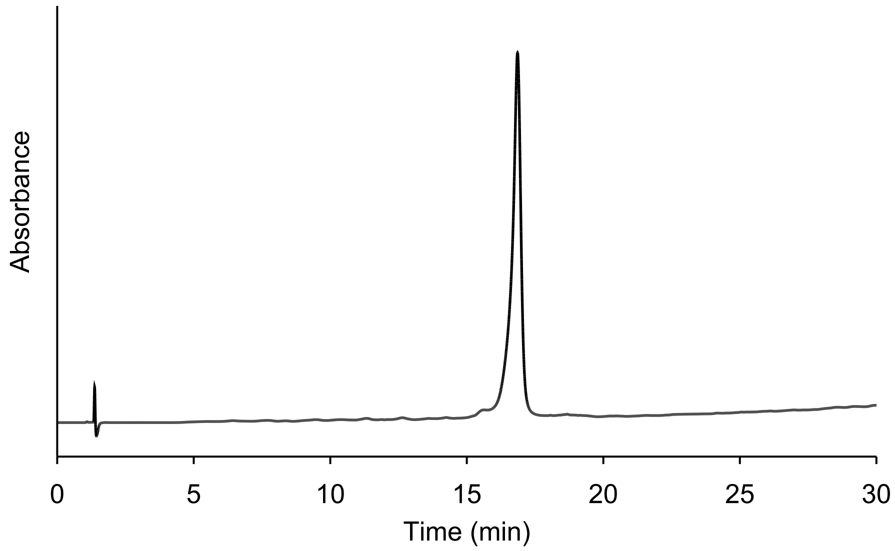

Peptoid 43 (*N*Lys*N*spe*N*spe)(*N*hArg*N*spe*N*spe)(*N*Lys*N*spe*N*spe)$_2$

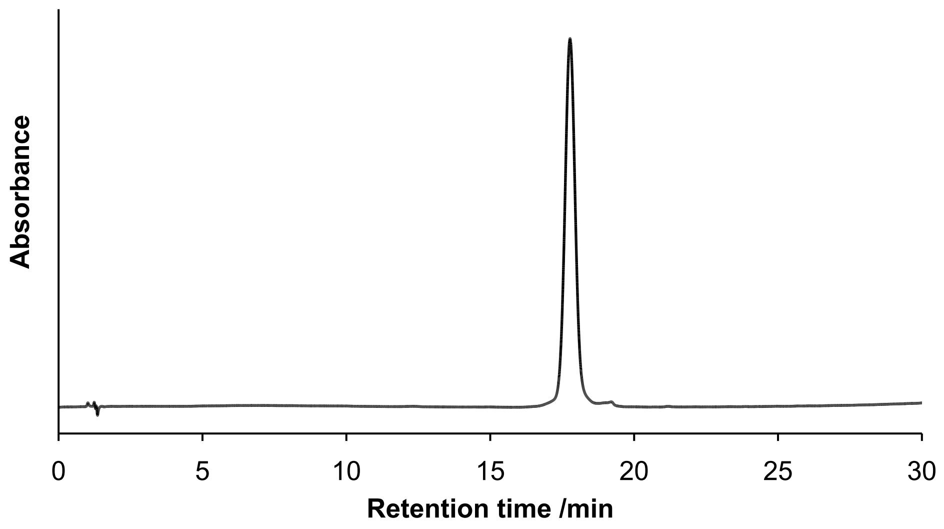

Peptoid 44 [(*N*hArg*N*spe*N*spe)(*N*Lys*N*spe*N*spe)]$_2$

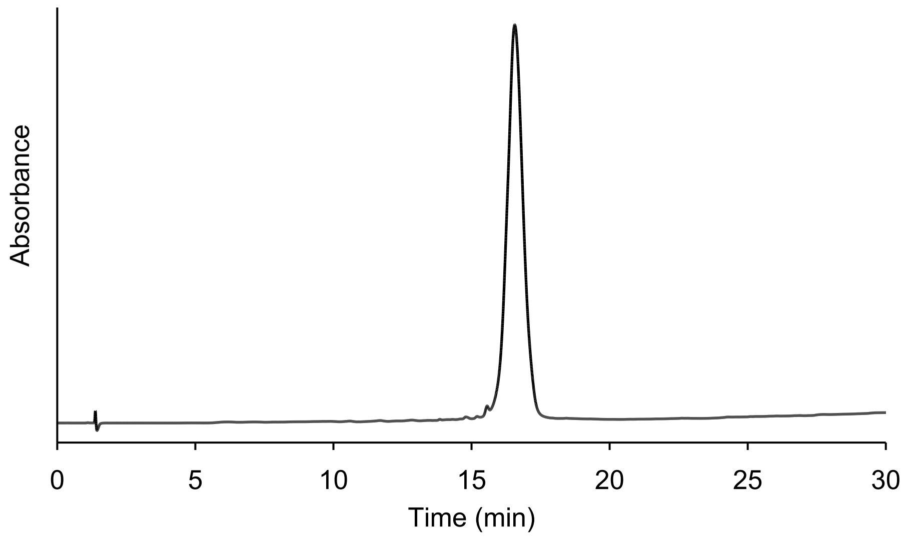

5. References

1. J.M. Andrews, *J. Antimicrob. Chemother.,* **2001**, *48*, 5.
